# Rapid gram-scale microwave-assisted synthesis of organic anodes for sodium-ion batteries with environmental impact assessment†

**DOI:** 10.1039/d4gc05530f

**Published:** 2025-01-06

**Authors:** Constantin Puscalau, Aamod V. Desai, Erlantz Lizundia, Romy Ettlinger, Mohamed Adam, Russell E. Morris, A. Robert Armstrong, Begum Tokay, Andrea Laybourn

**Affiliations:** a Advanced Materials Research Group, Faculty of Engineering, University of Nottingham Nottingham NG7 2RD UK; b EaStCHEM School of Chemistry, University of St Andrews North Haugh St Andrews KY16 9ST UK; c The Faraday Institution, Quad One Harwell Science and Innovation Campus Didcot UK; d Life Cycle Thinking Group, Department of Graphic Design and Engineering Projects. University of the Basque Country (UPV/EHU) Plaza Ingeniero Torres Quevedo 1 Bilbao 48013 Biscay Spain; e BCMaterials, Basque Center for Materials, Applications and Nanostructures, Edif. Martina Casiano Pl. 3 Parque Científico UPV/EHU Barrio Sarriena Leioa 48940 Biscay Spain; f TUM School of Natural Sciences, Department of Chemistry, Technical University of Munich Lichtenbergstrasse 4 85748 Garching Germany; g Institute of Process Research and Development &School of Chemistry, University of Leeds Leeds LS2 9JT UK A.Laybourn@leeds.ac.uk

## Abstract

Development of sustainable synthesis methods of organic electrode materials (OEMs) for sodium (Na)-ion batteries must take hold rapidly in large scale-synthesis if subsequent commercialisation is to occur. We report a facile and rapid gram-scale synthesis method based on microwave irradiation for disodium naphthalene-2,6-dicarboxylate (Na-NDC) and mono/disodium benzene-1,4-dicarboxylate (Na-BDC) as model compounds. Phase purity and formation of materials was confirmed by various characterisation techniques. The electrochemical performance was tested in both half and full cell formats and compared to material obtained *via* smaller scale synthesis, revealing state-of-the art performance in terms of capacity retention and cyclability. The environmental impacts upon organic anode synthesis were quantified according to *cradle-to-gate* life cycle assessment (LCA). The results allow for the identification of environmental hotspots during production, indicating areas for future process optimisation. Interestingly, remarkably reduced impacts are obtained compared to conventional syntheses at milligram scale. Additionally, this work suggests potential significant improvements upon additional upscaling and solvent recycling.

## Abstract 2

Gram-scale microwave synthesis and environmental impact assessment of sodium carboxylates for use as organic electrode materials in sodium-ion batteries.

## Graphical abstract

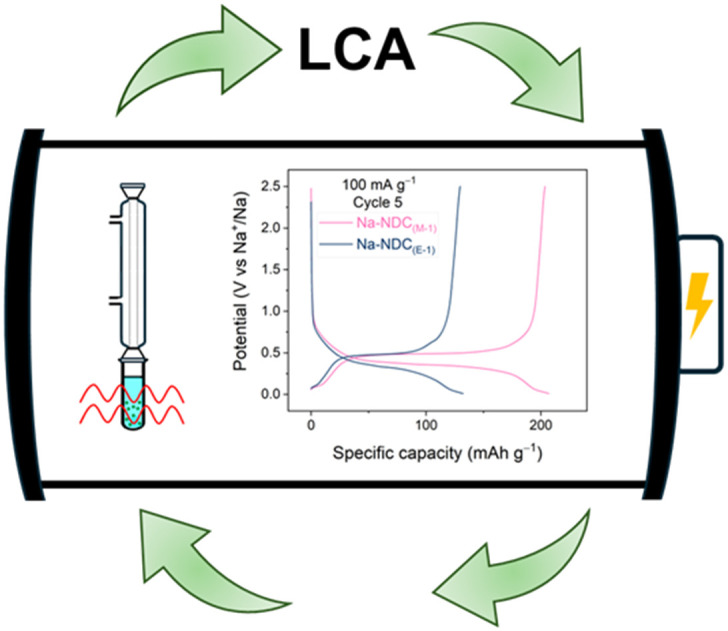

**Green foundation**

1. This work advances the sustainable synthesis of organic electrode materials for sodium (Na)-ion batteries by reporting (i) a new facile and rapid gram-scale synthesis based on microwave irradiation for disodium naphthalene-2,6-dicarboxylate (Na-NDC) and mono/disodium benzene-1,4-dicarboxylate (Na-BDC) and (ii) quantification of environmental impacts of their syntheses according to cradle-to-gate life cycle assessment (LCA).

2. Much reduced reaction times of 30 and 60 minutes in methanol and ethanol gave good yields close to 85% and 83% for Na-NDC and Na-BDC on the gram scale. Electrochemical performance is comparable to the materials obtained using conventional syntheses or smaller scale MW-assisted preparation. LCA results allow for the identification of environmental hotspots during production indicating areas for future process optimisation such as renewable resources and solvent recycling.

3. To model end-of-life of the new anodes using LCA, recycling or (bio)degradation experiments of the Na-BDC/Na-NDC anodes is required.

## Introduction

The increasing demand for battery storage systems over the past 20 years has seen the development of renewable and more efficient battery technologies. Lithium (Li)-ion batteries are the most commercially available systems today, operating in both mobile and stationary energy storage applications. Despite its widespread application, Li-ion technology is rapidly reaching its own limits in terms of energy density, with very few chemical design alternatives for substantial improvement.^1,2^ Na-ion batteries (SIBs) are regarded as a promising^3,4^ alternative to Li-ion batteries. The much higher Na abundance in Earth's crust than Li (2.3% and 0.002% respectively), and the lower price for SIBs over LIBs are desirable.^5^ In addition, current Li-ion battery performance relies heavily on electrode component materials that are mostly based on transition metals such as cobalt, an essential metal for the electrode design in LCO (lithium cobalt oxide, LiCoO_2_), and NMC (lithium–nickel–manganese–cobalt–oxide). Many ethical concerns have been raised in regard to the mining process of these metals,^6,7^ and some of these metals are at a high supply risk and have been assessed as critical raw materials (CRMs).^8^

Efficient design of anode materials remains necessary to overcome the slow kinetics and reduced rate of Na-ion diffusion in active materials, to facilitate the large-scale development and commercialisation of energy storage. Organic electrode materials (OEMs) offer several advantages over inorganic electrode materials, such as cost-efficiency, sustainability and natural abundance (*e.g.*, C, H, O, N, S), along with vast structural variety and tunability.^9^ For post-Li ion batteries, these benefits are particularly relevant for anode materials, which require development of suitable electrode materials.^10^ As the redox activity of organic anodes resides in the functional groups (azo, imine, carboxylate *etc*.) of the molecule,^11^ the versatility of molecular design can offer several advantages over pure inorganic materials, such as capacity improvement, cycling stability and coulombic efficiency. Owing to these advantages, many organic materials, especially those based on smaller building units, have started to be investigated in the past decade. In particular, aromatic carboxylate-based compounds show potential for conversion and storage of electrical energy; the carboxylate/carbonyl group can carry the redox activity, undergoing electron transfer through a reversible electrochemical reaction, whereas extended π-conjugated structures can also enhance the rate of charge transportation.^12^ Together with low molecular weights, the flexible structures can accommodate large ions, such as Na^+^, leading to faster redox reaction and superior capacities than pure inorganic anodes.^13^

Currently available synthesis methods of aromatic carboxylate compounds have limitations in terms of yield, as they rely on simpler design methods rather than efficient fabrication techniques. Commonly used synthesis methods, such as solvothermal, have the advantage of producing materials with a high degree of crystallinity, but require the use of solvents at relatively high temperatures (100–300 °C) and high pressures (up to 5000 atm).^14^ One pot synthesis at ambient temperature, on the other hand, can be an alternative. Nevertheless, it is important to note that this method can frequently lead to low yields and long reaction durations, often ranging from 24 to 48 h. Other issues encompass inadequate consistency between batches, extended reaction times, substantial amounts of hazardous solvent, and expensive scale-up processes accompanied by considerable energy usage.

Microwave-assisted synthesis is widely used in coordination chemistry for a variety of materials, including zeolites, covalent organic frameworks, and metal–organic frameworks.^15–19^ Microwave (MW) irradiation offers several advantages over conventional heating, including shortening reaction time (from hours to minutes compared to traditional solvothermal/hydrothermal processes), high yields and product conversion.^20^ Rapid heating in both nucleation and crystal growth stages leads to crystals with small narrow particle size distribution, homogeneous morphology and higher surface area, crucial properties for Na-ion anode materials.^21^ Hence, significantly decreased synthesis durations allow an increase in energy efficiency and a cut down of energy consumption, crucial to permit easy scale-up. While seeking larger scale synthesis adoption for less explored battery materials, standardised environmental impact metrics are urgently needed to prevent unexpected environmental impact transfer when adopting new battery chemistries that could replace existing electrochemical energy storage technologies.^22–25^ As such, it is possible to determine whether the development of new materials offers actual net improvements in environmental terms. Accordingly, the environmental impacts of Na-ion batteries can be accounted for by utilising the life cycle assessment (LCA) methodology, which follows the ISO 14040/44 international standards.^24,26^ LCA is already an essential tool for designing sustainable solutions in the energy field, both in research and at industry levels. When implemented early in the development process, LCA can identify areas for improvement and guide the design of new materials and batteries with better environmental performance.^27–29^ In addition to quantifying greenhouse gas emissions resulting from battery materials development, LCA determines multiple environmental impacts including the cumulative energy demand, acidification, ecotoxicity, eutrophication, or water consumption.^30^

Herein, we report a time-efficient and gram-scale microwave-assisted synthesis of sodium carboxylates, using disodium naphthalene-2,6-dicarboxylate (Na-NDC) and disodium benzene-1,4-dicarboxylate (Na-BDC) as model compounds. A synthesis protocol was operated using microwaves under reflux at atmospheric pressure and a study of impact of reaction rate and solvent (methanol and ethanol) on materials yield was conducted, along with detailed material characterisation and cycling studies to understand optimal synthesis conditions. In addition, the sustainability metrics of the syntheses have been calculated in detail.

## Experimental section

### Materials and methods

#### Chemicals

All chemicals and reagents were used as purchased without further purification: ethanol (absolute, SpS grade) was purchased from Scientific Laboratory Supplies Ltd; all water used was deionised. Methanol 99.8% was purchased from Sigma Aldrich. Sodium hydroxide (NaOH) was obtained from VWR Chemicals; benzene-1,4-dicarboxylic acid (99+%) and 2,6-naphthalenedicarboxylic acid (95%) from Sigma Aldrich. The chemicals for preparing electrodes were obtained commercially and used without any further purification. These include – conductive carbon (Super C65, Imerys Graphite & Carbon) and sodium carboxymethyl cellulose (CMC, degree of substitution 1.2, Sigma Aldrich).

## Experimental details

### Synthesis protocol

NaOH (0.8 g, 20 mmol), terephthalic acid (1.33 g, 8 mmol)/naphthalene-2,6-dicarboxylate (1.72 g, 8 mmol) and methanol/ethanol (32 mL) were mixed in a 60 mL borosilicate test tube and placed inside the cavity of a Sairem MiniFlow 200SS (Sairem, Neyron, France), where MW irradiation is produced by a solid-state generator at a 2.45 GHz frequency. The electric field was physically pre-matched to the reaction mixture using a stub tuner and sliding short circuit, in order to reduce the reflected power to a minimum. The glass tube was further connected to a reflux condenser, to avoid build-up of pressure and to operate in open-system conditions. The continuous control and monitoring of the forward power, the reflected power and temperature was achieved *via* an integrated PLC/touch screen digital display and the temperature measurement was performed *via* the built-in fibre optic thermometer. The microwave power, duration of microwave irradiation and temperature were controlled with a programmer. Working conditions were set at a target temperature (65 °C and 79 °C for methanol and ethanol, respectively, considering respective boiling points) and variable power, with different reaction times. After reaction completion, the hot solutions were allowed to cool naturally at room temperature and centrifuged for solids collection and washed with fresh solvent (Tables S1 and S2†). Finally, the products were dried overnight at 60 °C.

### Structural characterisation

Powder X-ray diffraction (PXRD) measurements of the materials were obtained using a Bruker D8 Advance DaVinci. Data were collected between 4° and 70° 2*θ* using Cu Kα radiation (*λ* = 0.15406 nm), step size 0.02° and step time 0.2 s. Pattern matching, and semi-quantitative analyses of constituents were performed with Profex 5.0 software. Simulated data were obtained from Mercury 4.1.3 with powder diffraction files from the databases (Na_2_NDC,^1^ NaHBDC: CCDC 226109, Na_2_BDC: CCDC 145817). Fourier-transform infrared (FT-IR) spectra were recorded on a Bruker platinum alpha FTIR spectrometer on the neat compound using the attenuated total reflection (ATR) technique. Scans were conducted between 500 and 4000 cm^−1^ and data plotted using OPUS 5.5 software. Material decomposition data were gathered by TGA using a TA Instruments TGA 550. Samples were heated at 10 °C min^−1^ under nitrogen atmosphere to a maximum temperature of 850 °C. Scanning electron microscope (SEM) images of all materials were recorded on a JEOL JSM-IT800 microscope. The measurement was done on powdered samples which were placed on copper tape. Multi-element analysis by ICP-MS of diluted solutions was undertaken by ICP-MS (Thermo Fisher Scientific iCAP-Q; Thermo Fisher Scientific, Bremen, Germany). Samples were introduced (flow rate 1.2 mL min^−1^) from an autosampler (Cetac ASX-520) incorporating an ASXpress™ rapid uptake module through a perfluoroalkoxy (PFA) Microflow PFA-ST nebuliser (Thermo Fisher Scientific, Bremen, Germany). Sample processing was undertaken using Qtegra™ software (Thermo Fisher Scientific) utilising external cross-calibration between pulse-counting and analogue detector modes when required. The instruments are run employing several operational modes. The iCAP-Q employs in-sample switching between two modes using a collision cell (i) charged with He gas with kinetic energy discrimination (KED) to remove polyatomic interferences and (ii) using H_2_ gas as the cell gas. Typically, in-sample switching is used to measure Se in H_2_-cell mode and all other elements in He-cell mode. Peak dwell times were 100 ms for most elements with 150 scans per sample. Internal standards, used to correct for instrumental drift, were introduced to the sample stream on a separate line (equal flow rate) *via* the ASXpress unit or are added directly to calibration standards and samples and introduced on a single line. Internal standards typically included combinations of Sc (10 μg L^−1^), Ge (10 μg L^−1^), Rh (5 μg L^−1^), Re (5 μg L^−1^) and Ir (5 μg L^−1^). The matrices used for internal standards, calibration standards, and sample diluents were typically 2% Primar grade HNO_3_ (Fisher Scientific, UK) with 4% methanol (to enhance ionisation of some elements). Calibration standards typically included (i) a multi-element solution with Ag, Al, As, Ba, Be, Cd, Ca, Co, Cr, Cs, Cu, Fe, K, Li, Mg, Mn, Mo, Na, Ni, P, Pb, Rb, S, Se, Sr, Ti, Tl, U, V, and Zn, in the range 0–100 μg L^−1^ (0, 20, 40, 100 μg L^−1^) (Claritas-PPT grade CLMS-2 from SPEX Certiprep Inc., Metuchen, NJ, USA); (ii) a bespoke external multi-element calibration solution (PlasmaCAL, SCP Science, France) with Ca, Mg, Na, and K in the range 0–30 mg L^−1^ and (iii) a mixed phosphorus, boron and sulphur standard made in-house from salt solutions (KH_2_PO_4_, K_2_SO_4_ and H_3_BO_3_).

### Electrochemical characterisation

The working electrodes for all materials were composed of the active phase (60 wt%), conductive carbon (30 wt%) and the binder (CMC, 10 wt%). These ratios are based on previously optimised studies on organic anode materials and are required to accommodate the low electronic conductivity of sodium carboxylates.^31^ The slurry was prepared by grinding the active material with carbon and adding that to a solution of CMC in water. The mixture was stirred for *ca.* 3 h and cast on to an Al-foil (Advent Research Materials) and air dried for 2 h. Electrode discs were punched (diameter 12 mm) and dried overnight under vacuum at 80 °C. The average mass loading of the samples was approximately 1.71 mg cm^−2^ and 1.88 mg cm^−2^ for Na-BDC_(M-1)_ and Na-NDC_(M-1)_ respectively. The cycling studies in both half-cell and full-cell formats were performed using coin cells (CR2032) with Na metal (Sigma Aldrich) as the counter electrode for half-cells. Glass fiber (Whatman GF/F) was used as the separator, and NaPF_6_ in EC (ethylene carbonate) and DEC (diethyl carbonate) (1 : 1, v/v) (Kishida Chemical, Japan) functioned as the electrolyte. For full-cells, the cathode material – Na_(0.79±0.05)_Ni_(0.27±0.05)_Mn_(0.42±0.05)_Mg_(0.15±0.05)_Ti_(0.17±0.05)_O_(2±0.05)_, which contained 92 wt% active, 3 wt% carbon, and 5 wt% binder was used as supplied on a C-coated Al foil. The cell assembly was carried out in an Argon-filled glovebox (MBraun) with O_2_ and H_2_O levels <1 ppm. The electrochemical cycling was performed at 30 °C from 0.01–2.5 V on a Biologic BCS-805 modular battery testing system or a Neware BTS tester. Data analysis was performed using BT-Lab or BTSDA software. The current rates and capacities are expressed based on the mass of the active materials, except in a cell containing only carbon and binder, where the calculations were based on the mass of carbon. For full-cells, the calculations were based on the active mass (AM) of anodes (Na-BDC or Na-NDC) and denoted as AM-anode.

### Life cycle assessment

The environmental impacts of synthesising Na-BDC and Na-NDC were evaluated using the life cycle assessment (LCA) methodology as set by the ISO 14040/44 international standards.^32^ The evaluation was conducted according to a *cradle-to-gate* system boundary. To that end, the assessment includes the raw material acquisition and transportation to the factory, the energy consumption during on-site synthesis, and the resulting waste management. During anode modelling, the end-of-life has been disregarded as a common procedure in emerging battery technologies due to lack of data regarding recycling or degradability. The impacts of the capital stock, *i.e.* the emissions caused by manufacturing the installation were not considered. For the End-of-Life (EoL) management, residual hazardous material landfill and hazardous waste incineration in Europe (without Switzerland) is considered. The calculations are based on the electricity available for low voltages grid (<1 kV) in the United Kingdom (sold at 0.107 € per kWh).

The energy consumption was estimated considering the maximum power of each instrument, functioning at a 70% workload, and taking into account the volumetric capacity of the instrument to process several samples together. The process inventory was retrieved from primary sources (our own laboratory data) and has been combined with a literature search (secondary data) to compare the results with five conventional syntheses. The assessment was conducted using the OpenLCA 2.1.1 software and the ecoinvent v3.10 cut-off processes database. Environmental impacts were determined using the ReCiPe 2016 Midpoint (H) methodology.

## Results and discussion

### Microwave-assisted synthesis

Na-NDC and Na-BDC are promising anode materials, with a two-electron redox reaction and a reversible (de)insertion mechanism for two Na^+^ cations.^33^ Syntheses of Na-NDC and Na-BDC were carried out by reacting the respective dicarboxylic acids with sodium hydroxide under reflux using microwave irradiation. The reaction involves deprotonation of the carboxylic acid, aided by an aqueous NaOH basic solution, additionally acting as the alkali metal source. Experimentally, all precursors are mixed inside a test tube, subsequently placed inside the cavity of a solid-state MW generator and reactions conducted under microwave irradiation with continuous stirring, generally offering yields >75% and an amount of material >1.2 g, with the highest reported yield (90%) corresponding to Na-NDC material produced under 1 h in ethanol. Overall, both methanol and ethanol solvents offered good results in terms of yield (Fig. S5†).

As for Na-BDC, the highest yield and amount of material obtained was for the reaction conducted in methanol for 30 min (81.9% and 1.38 g respectively), whereas the same reaction time with ethanol gave a 74.5% yield (1.26 g of material). A comparison of yield achieved by our MW-assisted method can be made with Abouimrane's work,^34^ where they managed to obtain yields for NaHBDC up to 62%, significantly lower than yields reported in this work. Syntheses conducted for 1 h presented similar yields in both solvents (81.5%). Overall, results indicate that reaction completion happens in a relatively short period of time, around 30 min, not increasing further with MW irradiation and reaction duration. As for Na-NDC, both synthesis in methanol and ethanol with 30 min reaction time and methanol 1 h accorded similar yields around 86%, whereas synthesis conducted in ethanol for 1 h showed an increased yield of 90%. Absence of reported yields for Na-NDC synthesis made comparison impractical with previous studies. Prior reported synthesis procedure of Na-NDC material employed stirring the linker and NaOH salt in methanol under reflux condition at 80 °C for 24 h;^35^ seemingly, with MW irradiation the reaction time can be drastically reduced to 1 h, still affording high yields (>85%).

Both Na-NDC and Na-BDC materials crystallize in a monoclinic space group *P*2_1_/*c*, however a monodeprotonated hydrogen terephthalate phase is also present for the latter, with a triclinic *P*1̄ space group and verified as the primary synthesis product. The structure of the synthesised Na-NDC materials was confirmed by PXRD diffraction patterns (Fig. S1b and S6†) and compared with the simulated structure. All experimental patterns show minor differences in peak widths and positions, fittingly matching the simulated pattern and confirming Na-NDC phase purity. As for Na-BDC materials, upon reaction between terephthalic acid and NaOH, full deprotonation of the linker is expected, thus obtaining Na_2_BDC, with its own characteristic simulated pattern (Fig. S1a and S8†). However, another phase, corresponding to the monosodium terephthalate salt may form due to partial deprotonation of H_2_BDC during synthesis, with the corresponding pattern highlighted in pink in Fig. S1a.† In both syntheses with methanol and ethanol, the good match between experimental patterns and simulated confirms that the major phase observed is NaHBDC, with minor presence of Na_2_BDC, due to appearance of very low intensity peaks characteristic of the disubstituted salt at 2*θ* = 31.5° and 38°. Na_2_BDC is highly soluble in water (13% (w/w)),^36^ whereas NaHBDC is only moderately soluble (terephthalic acid has relatively low p*K*_a_ values: 3.51, 4.82). A possible hypothesis for the formation of NaHBDC is that when Na_2_BDC is formed, the salt may begin to convert into the monosubstituted species due to its moderate solubility in water (solvents used contain *ca*. 0.2% water). This conversion could potentially shift the equilibrium between Na_2_BDC and NaHBDC towards the monosubstituted species, which could be further enhanced, particularly with longer reaction times.

From Fig. S1d and S7,† both solvents utilised during Na-NDC syntheses gave samples with similar FT-IR spectra. The absence of a broad O–H stretching peak in the range 3250–2000 cm^−1^, characteristic of carboxylic acids present in the parent naphthalene-2,6-dicarboxylic acid, confirmed salt formation. Additionally, the pure naphthalene linker showed an intense band corresponding to carbonyl (–C=O) stretching at 1685 cm^−1^, noticeably blue shifted and split into two bands in the Na-NDC spectra (at 1557 cm^−1^ and 1395 cm^−1^ respectively, which were assigned to asymmetric and symmetric stretching vibrations).

FT-IR spectra of Na-BDC materials showed a band at 1675 cm^−1^ assigned to carboxylic acid –COO vibrations, originating either from unreacted terephthalic acid starting material, or from the non-coordinated carboxylic acid in the monosodium terephthalate (Fig. S1c and S9†).^37^ Due to resonance stabilisation, terephthalic acid has relatively low p*K*_a_ values (3.51, 4.82),^38^ which allow easy deprotonation by NaOH, affording mono/di-sodium terephthalate through the acid–base chemistry. Spectra of both salts indicate metal carboxylate formation; the bands at 1551 cm^−1^ and 1380 cm^−1^ were assigned to *ν*_as_ (COONa) and *ν*_s_ (COONa), two characteristic fingerprints of salt formation. SEM images of Na-NDC material synthesised in methanol presents small particles (1–8 μm) with irregular morphology (Fig. S2†). Syntheses conducted in ethanol gave morphologically non-uniform size and shape, with confined aggregates. Na-BDC with ethanol exhibited submicron particles along with bigger crystals (5–10 μm), whereas in methanol, the salt presents again large agglomerates and irregular shape.

To quantify product formation and distinguish between starting unreacted linker and salt, ICP-MS analysis was performed for cross-analysis with CHN (elemental analysis) with results presented in Table S4.† Based on H % of Na-BDC samples for syntheses conducted in both methanol and ethanol solvents (2.02 and 2.58% respectively), Na mono/di-substituted salt is obtained as the main product: even though Na-BDC in ethanol showed the highest hydrogen content (2.58%), Na presence is half of the expected Na_2_BDC (10.81% and 21.88% respectively), suggesting less of the disubstituted salt formation, but large presence of NaHBDC (*ca.* 49%) and unreacted linker, confirmed by PXRD (Fig. S1a†) and by TGA (Fig. S3a†). As for syntheses conducted in methanol, 9.86% of Na suggests even more presence of unreacted linker. Na-NDC materials, on the other hand, showed lower hydrogen wt% than theoretical Na_2_NDC (2.32 wt%), 1.92% and 1.75% for syntheses conducted in methanol and ethanol respectively, indicating successful formation of disubstituted salt as main reaction product. Confirmation of Na_2_NDC presence was further given by ICP analyses, showing wt% of Na very close to expected Na_2_NDC (17.67%), with 16.59% for methanol and 17.52% for ethanol.

Overall, ICP-MS results support CHN results for more Na content in Na-NDC samples than Na-BDC, the former accounting for more presence of NaHBDC rather than Na_2_BDC. Thermogravimetric analyses of as-synthesised materials is presented in Fig. S3.† TGA profiles of Na-NDC samples showed no significant weight loss until around 520 °C, indicating high temperature of decomposition of materials likely due to efficient π-stacking interaction between adjacent naphthalene rings.^39^ Na-NDC in ethanol presented a 2% weight loss at around 350 °C, associated with unreacted linker decomposition, whereas in methanol less than 1% weight loss was observed. Final wt% loss of both samples (64% for products produced in ethanol and 73% for methanol) indicate reduced presence of expected salt formation for methanol-synthesised material, thus validating CHNS/ICP-MS analyses.

For Na-BDC samples, a significant weight loss at around 300 °C was observed for methanol and ethanol samples (53.2% and 53% respectively), due to unreacted linker, however comprised of 30.4% linker from in-pore NaHBDC structure. Overall, both TGA profiles are very similar, presenting a step above 520 °C associated with the loss of coordinated ligand and decomposition of the salt.^40^ The good match between the two TGA curves is in accordance with previous PXRD and FT-IR analyses discussions which evidence NaHBDC formation as the main product.

### Electrochemical studies

**Fig. 1:**
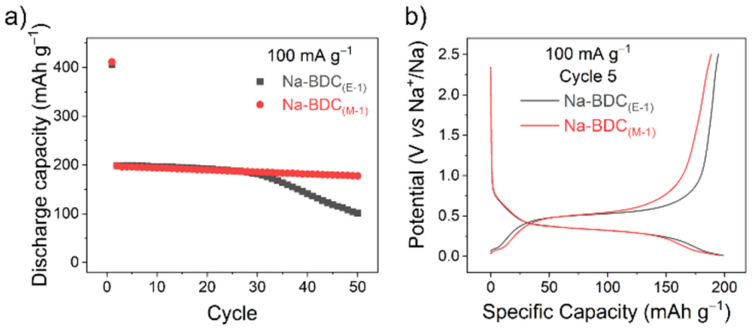
(a) Discharge capacity and (b) galvanostatic charge/discharge curves for Na-BDC materials (5th cycle) cycled between 0.01–2.5 V at a current rate of 100 mA g^−1^.

To further screen the materials electrochemically, initial tests were performed on half-cells with sodium metal as the counter electrode. Electrodes of all materials were prepared using a water-soluble binder (CMC – carboxymethyl cellulose, 10 wt%), in a composition containing 60 wt% active material and 30 wt% conductive carbon (Super C65). Galvanostatic cycling was performed at a current rate of 100 mA g^−1^, where Na-BDC_(M-1)_, Na-BDC_(E-1)_ showed high first discharge capacities (∼405 mA h g^−1^), (Fig. 1a).

**Fig. 2:**
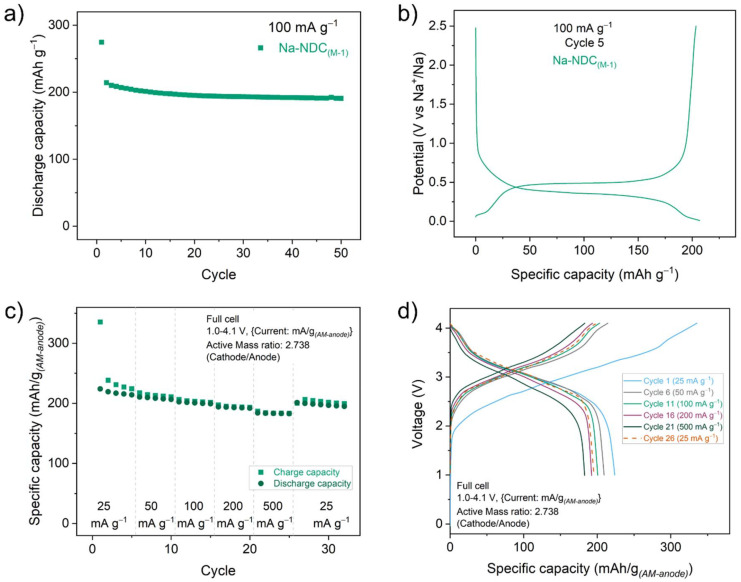
(a) Discharge capacity and (b) galvanostatic charge/discharge curves for Na-NDC materials (5^th^ cycle) cycled between 0.01–2.5 V at a current rate of 100 mA g^−1^. Rate performance for full-cell prepared using Na-NDC_(M-1)_ as anode, (c) discharge capacity at different current rates, cycled five times at every step, and (d) corresponding charge/discharge curves for first cycle of every step.

A significant loss of reversible capacity was observed during the first desodiation of the Na-BDC_(E-1)_ material (ICE values ∼43–45%), which could partly be ascribed to the formation of a solid–electrolyte interface (SEI) layer^41^ and to ion-insertion in the conductive carbon additive (∼37 mA h g^−1^; Fig. S10†). Notably, previous studies on these materials have seen lower ICE values as well, with 30% for H_2_BDC,^42^ 40% for NaHBDC,^34^ and 50 to 60% for Na_2_BDC.^40,43^ In both cases a single step redox profile was observed, suggesting the absence of large amounts of unreacted linker or any side-reactions during the MW-assisted synthesis (Fig. 1b). The discharge capacities from the 2^nd^ cycle onward stabilised at ∼200 mA h g^−1^ for both Na-BDC_(M-1)_ and Na-BDC_(E-1)_. Na-BDC_(M-1)_ showed minimal loss in capacity with a discharge capacity of 178 mA h g^−1^ after 50 cycles. A similar experiment in the case of Na-NDC suggested the superior performance for Na-NDC_(M-1)_ over Na-NDC_(E-1)_ (Fig. 2a), and S11†). This performance for Na-NDC_(M-1)_ is comparable to the cycling capacities observed for the material obtained on small scale synthesis (Fig. S12†).^39^ Na-NDC materials showed stable capacity retention over 50 cycles with high ICE values of 77% and 67% for Na-NDC_(M-1)_ and Na-NDC_(E-1)_, respectively.

Like in the case for Na-BDC, Na-NDC exhibited a single plateau at ∼0.35 V in the discharge profiles, further endorsing its clean formation (Fig. 2b). Although Na-NDC_(E-1)_ exhibited a similar voltage trace, the discharge capacities were lower, suggesting the influence of trace amounts of unreacted precursor during the synthesis. Parent carboxylic acids can offer charge storage ability, however, the Na-ion-insertion is associated with liberation of gases, lower initial coulombic efficiencies (ICE), and large structural changes, which affect the cycling performance.^34,42^

Based on the results for half-cells, Na-BDC_(M-1)_ and Na-NDC_(M-1)_ were chosen for testing in full-cells *vs.* oxide-based cathode. For reference, half-cell cycling profiles for the cathode material are shown in Fig. S13.† Full cells containing Na-(BDC)_(M-1)_ exhibited a high first cycle discharge capacity of 245 mA h g_(AM-anode)_^−1^, with relatively stable capacities over 100 cycles (Fig. S14a†). A significant loss of reversible capacity was observed for the 1^st^ charge (Fig. S14b†), which can be linked to the poor ICE that was observed for half cells of Na-BDC compounds. In the case of full-cell measurements involving Na-NDC_(M-1)_ much better reversibility was observed, with a first discharge capacity of 214 mA h g_(AM-anode)_^−1^ (Fig. S15†). Also, this capacity was reasonably retained with discharge capacity of 146 mA h g_(AM-anode)_^−1^ at the end of 100 cycles (Fig. S15a†).

Encouraged from this performance and to further understand the full cell behaviour of Na-(NDC)_(M-1)_, a rate capability test was carried out at increasing current rates up to 500 mA g_(AM-anode)_^−1^ (Fig. 2c and d). The fading of capacity at varying current densities was minimal, with a discharge capacity of ∼184 mA h g_(AM-anode)_^−1^ at the current density of 500 mA g_(AM-anode)_^−1^. The specific capacity at the initial cycles could be regained when the current was returned to 25 mA g_(AM-anode)_^−1^.

### Environmental impact assessment

**Fig. 3:**
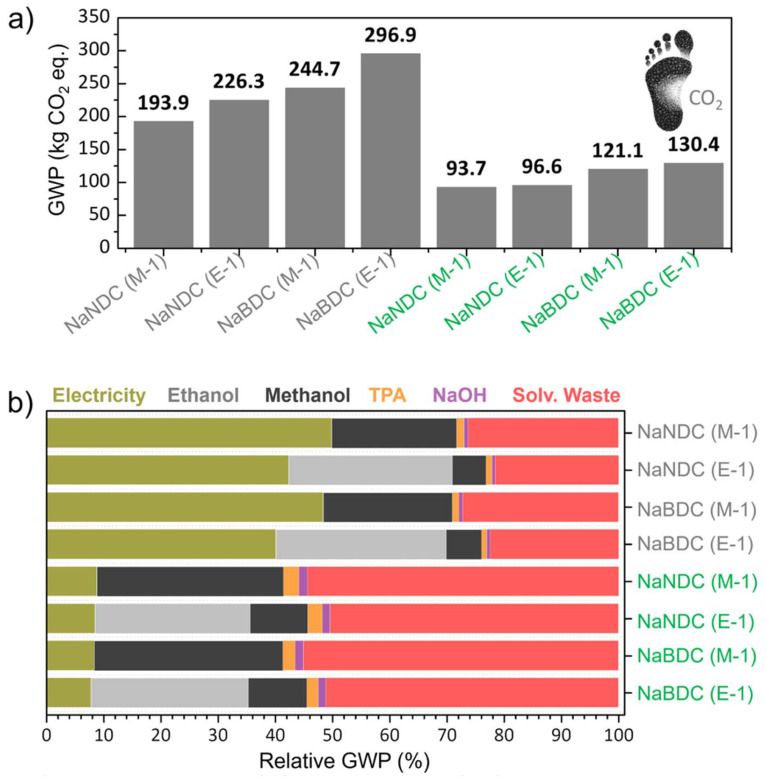
Global warming potential (GWP) of Na-NDC and Na-BDC. (a) Absolute value and (b) relative CO_2_ share depending on process input/outputs. Green labels represent syntheses performed utilising a renewable energy grid (“*market for electricity, low voltage, renewable energy products* | *electricity, low voltage, renewable energy products | Cutoff, U – CH*”). TPA = terephthalic acid/H_2_BDC.

Incorporating redox-active organic material into anodes, instead of non-renewable inorganic materials, aligns well with Green Chemistry principles and circularity targets. To determine whether the materials synthesised herein offer clear environmental sustainability benefits, a life cycle assessment (LCA) was conducted. A *cradle-to-gate* system boundary was used to account for the impacts generated during raw material acquisition, transportation to the factory gate, energy consumption during processing, and waste management. To ensure transparency and enable future comparison, the complete input and output data, the life cycle inventory (LCI), for the organic anodes is disclosed in the flowcharts in Fig. S16–S19 and Tables S5–S8.† Despite the low technology readiness level (TRL) of the analysed anode syntheses processes, this study is useful as it provides initial insights into the environmental sustainability of materials obtained in the early stages of development.^44,45^Fig. 3a shows the 100-year time horizon global warming potential (GWP) of the Na-NDC and Na-BDC syntheses, whose value ranges from 193.9 and 296.9 kg CO_2_-equiv. per kg of synthesised material. Overall, Na-BDC has larger impacts over Na-NDC because of reduced output quantities (1.38 *vs.* 1.88 g, respectively) for comparable input amounts.

As a result, it remains evident that increasing the processing scale yields significant environmental benefits, particularly in terms of energy utilisation. Furthermore, E-1 syntheses bear a larger burden (17–21% increase) compared to M-1. To provide context for the obtained CO_2_ footprint, we further studied the GWP of five conventional (non-microwave) synthetic routes for these materials reported in the literature.^35,40,46,47^ The life cycle inventory was obtained from secondary sources (published manuscripts) and is disclosed in the ESI as Fig. S20–S24 and Tables S9–S13† (data uncertainties exist in those processes as obtained from secondary sources, so these results should be only considered as a guidance). GWP values of 3845 to 11 390 kg CO_2_-equiv. kg^−1^ are obtained for Na-NDC, while Na-BDC shows values of 175–796 kg CO_2_-equiv. kg^−1^ (Fig. S25†). Therefore, it remains evident that the synthetic procedures developed herein bear significant environmental benefits over conventional procedures for Na-NDC and Na-BDC anodes, which rely on the utilisation of long reactions (such as, up to 24 h and 90 °C under magnetic stirring) with a large associated energy consumption. As a matter of fact, ∼41 kWh is computed per g of material for the stirring step in Na-NDC (Conv. 1), significantly larger than the ∼0.33 kW h required for the microwave-assisted synthesis of Na-NDC_(M-1)_. When looking into the literature on biomass-derived hard-carbon anodes for Na-ion batteries, Trotta *et al.* reported GWP values of 500 and 615 kg CO_2_-equiv. kg^−1^ for Kuranode-derived and glucose-derived hard carbon anodes respectively,^48^ while Liu *et al.* disclosed values of 167–3143 kg CO_2_-equiv. kg^−1^ for bamboo, pine, coconut, tea seed, hazelnut shell, and peanut shell-derived hard carbons.^49^ Therefore, it remains evident that the organic anodes developed herein by MW heating render clear environmental benefits over carbonaceous alternatives.

In this context, and in light of the current global decarbonisation efforts of the power sector towards carbon emissions mitigation,^50^ assessing the CO_2_ footprint using a fully renewable energy mix could provide valuable information on the actual impacts in a near future scenario. In this context, biorefinery platforms are also increasingly relevant in the current industrial sector as they can deliver bio-based chemicals and materials at a reduced footprint.^51^ Accordingly, we implemented a low-voltage renewable energy, methanol from biomass origin and ethanol from fermentation (instead of fossil-based), and the updated GWP value is also given in Fig. 3a. The values of 93.7–130.4 kg CO_2_-equiv. kg^−1^ are obtained, which represents a 52–56% impact reduction. Therefore, it remains evident that energy consumption is one of the largest drivers in terms of CO_2_ emissions for the materials developed herein. To gain further insights on Na-NDC and Na-BDC syntheses, the disaggregated GWP values are provided in Fig. 3b. When utilising a fossil-based energy grid, the *cradle-to-gate* CO_2_ emissions are primarily caused by electricity consumption (40.1-to-49.9%), followed by spent solvent use during syntheses and spent solvent waste-treatment. On the contrary, the use of the monomer and NaOH has a contribution below 2% to the total GWP. This can be explained by the comparatively lower quantities of NaOH, and monomer utilised over solvents (note that in general 96 mL solvent is needed to process <2 g of monomer with the addition of <1 g of NaOH). After shifting to the renewable electricity grid, energy contribution requirements are lowered to ∼8%, while the treatment of generated solvent waste accounts for 50–55% of the total GWP. Overall, these findings indicate a crucial role of energy consumption and solvent-waste treatment in determining the environmental impact of Na-NDC and Na-BDC organic anodes.

**Fig. 4:**
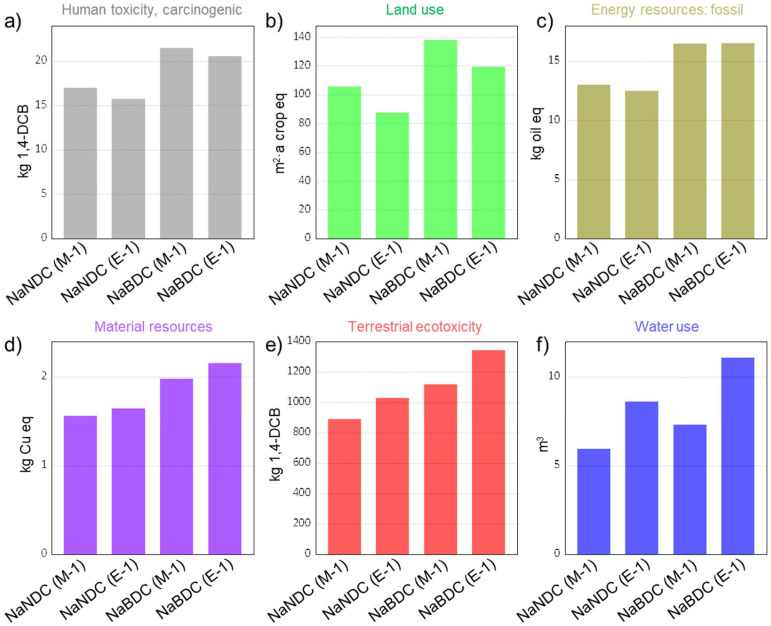
*Cradle-to-gate* environmental impacts in six categories normalised to 1 kg of material. Impact categories include: (a) human toxicity, carcinogenic; (b) land use; (c) energy resources, fossil; (d) material resources: metals/minerals; (e) terrestrial ecotoxicity; and (f) water use.

To gain a comprehensive understanding, we also considered other relevant environmental impact metrics, including *human toxicity (carcinogenic)*, *land use*, *energy resources use (fossil)*, *material resources use (metals*/*minerals)*, *terrestrial ecotoxicity*, and *water consumption*. Results are summarised in Fig. 4 (see Tables S14–S16† for extended information). Overall, the same trend as previously observed for the impact category of GWP is now visible as well. However, certain changes could be detected depending on the impact category. In this context, with a value of 21.49 kg 1,4-DCB-equiv., the Na-BDC_(M-1)_ shows the largest impact in the category of *human toxicity (carcinogenic)*. This large value originates from the use of methanol from biomass gasification (43% share), and electricity consumption (40% share). A similar trend is achieved in *land use* and *energy resources use (fossil)*, where methanol is responsible for 96% and 64% of the impacts in those categories, respectively. These results indicate that, in spite of the preferable character of methanol *vs.* ethanol when considering health risks,^52^ such advantages are not directly translated into reduced *cradle-to-gate* environmental impacts, which consider the whole supply chain impacts until processing. The impact categories of material resources and terrestrial ecotoxicity show a comparable trend with GWP, with values of 1.56–2.15 kg Cu-equiv. and 889–1342 kg 1,4-DCB-equiv., respectively. In terms of *water use*, E-1 syntheses render larger impacts. Such larger water consumption is attributed to use of ethanol sourced from biomass fermentation (sugar cane, sugar beet, maize, grass, rye, potatoes, wood, whey, sweet sorghum), which holds upstream water needs. Overall, these results indicate that the NaNDC_(M-1)_ anode offers the lowest environmental impacts when considering seven different categories.

Once the end-of-life (EoL) of the anodes has been reached, these materials can be returned to the battery supply chain through recycling by established pyrometallurgical or hydrometallurgical processes, or through the more innovative direct recycling. However, in the absence of appropriate infrastructure, inorganic anodes will eventually end up in incinerators or landfills, adding to the environmental burden of these materials. According to LCA (ecoinvent v3.10; ReCiPe 2016 Midpoint (H)),^53^ the end-of-life management of hazardous waste under landfill and incineration (no energy recovery) scenarios bear a GWP of 3.40 × 10^−3^ and 2.42 kg CO_2_ per kilogram. In this context, organic anode materials offer significant EoL sustainability benefits as they are safer (avoiding undesired toxic material leaching out during landfill) and could be potentially biodegraded into non-toxic by-products under industrial composting conditions, closing the Earth's carbon cycle.

## Conclusions

In summary, disodium naphthalene-2,6-dicarboxylate and disodium benzene-1,4-dicarboxylate were successfully synthesised at gram-scale under microwave-assisted synthesis. Reaction times of 30 and 60 minutes in methanol and ethanol offered good yields close to 85% and 83% for Na-NDC and Na-BDC respectively. For Na-BDC materials, monosubstituted salt (NaHBDC) was the main product of the synthesis with both solvents, whereas the fully disubstituted salt was the main product for naphthalene-based material (Na_2_NDC). Product formation was further investigated by different analysis techniques. Both the materials were tested in half- and full-cell formats, with comparable performance observed (in terms of specific capacities, cycling stability, and ICE values) to the materials obtained using conventional syntheses or smaller scale MW-assisted preparation. Life cycle assessment results indicate a *cradle-to-gate* global warming potential of 93.7–130.4 kg CO_2_-equiv. kg^−1^ for the Na-NDC and Na-BDC anode materials when renewable resources are implemented. Moreover, the Na-NDC_(M-1)_ anode, synthesised using methanol, offers the lowest environmental impacts when considering all the impact categories. Overall, this work demonstrates that microwave irradiation can be a powerful tool for rapid and energy-efficient syntheses of carboxylate-based organic compounds in good yields and gram scale, with satisfactory structural and electrochemical features for implementation as anode materials for Na-ion batteries, offering opportunities for scale-up methods and practical implementation.

## Author contributions

Constantin Puscalau: conceptualization, data curation, formal analysis, investigation, methodology, visualization, writing – original draft, writing – review & editing. Aamod V. Desai: conceptualization, data curation, formal analysis, investigation, methodology, visualization, writing – original draft, writing – review & editing. Erlantz Lizundia: data curation, formal analysis, investigation, software, visualization, writing – original draft, writing – review & editing. Romy Ettlinger: data curation, formal analysis, investigation, visualization, writing – review & editing. Mohamed Adam: data curation, formal analysis, investigation, methodology, visualization, writing – review & editing. Russell E. Morris: methodology, resources, project administration, supervision, writing – review & editing, funding acquisition. A. Robert Armstrong: methodology, resources, project administration, supervision, writing – review & editing, funding acquisition. Begum Tokay: resources, supervision, writing – review & editing. Andrea Laybourn: conceptualization, methodology, resources, project administration, supervision, writing – review & editing, data curation, funding acquisition.

## Data availability

Data for this article, including raw datasets for PXRD patterns, ICP-MS, FTIR spectra, TGA, and SEM images, electrochemical data for half- and full-cells are available at the University of Nottingham Research Data Repository, https://doi.org/10.17639/nott.7502. Data supporting this article (including datasets for LCA) are included as part of the ESI.†

## Conflicts of interest

There are no conflicts to declare.

## Supplementary Material

GC-027-D4GC05530F-s001
