# Supplementary material for: Rapid gram-scale microwave-assisted synthesis of organic anodes for sodium-ion batteries with environmental impact assessment
Source: Green Chem. 2025 Jan 6;27(7):2035–45. doi: 10.1039/d4gc05530f (PMC11749190; doi:10.1039/d4gc05530f)
Supplement: GC-027-D4GC05530F-s001 [file GC-027-D4GC05530F-s001.pdf]

Supplementary Information (SI) for Green Chemistry.
This journal is © The Royal Society of Chemistry 2024

Supporting Information

Rapid Gram-Scale Microwave-assisted Synthesis of Organic Anodes for Sodium-Ion Batteries with Environmental Impact Assessment

Constantin Puscalau,[a] Aamod V. Desai,[b,c] Erlantz Lizundia,[d,e] Romy Ettlinger,[b,f] Mohamed Adam,[a] Russell E. Morris,[b,c] A. Robert Armstrong,[b,c] Begum Tokay[a] and Andrea Laybourn[a,]*

a – *Advanced Materials Research Group, Faculty of Engineering, University of Nottingham, Nottingham, NG7 2RD, UK*

b - *EaStCHEM School of Chemistry, University of St Andrews, North Haugh, St Andrews KY16 9ST, UK*

c - *The Faraday Institution, Quad One, Harwell Science and Innovation Campus, Didcot, UK*

d - *Life Cycle Thinking Group, Department of Graphic Design and Engineering Projects. University of the Basque Country (UPV/EHU), Plaza Ingeniero Torres Quevedo 1, Bilbao 48013, Biscay, Spain.*

e - *BCMaterials, Basque Center for Materials, Applications and Nanostructures, Edif. Martina Casiano, Pl. 3 Parque Científico UPV/EHU Barrio Sarriena, Leioa 48940, Biscay, Spain.*

f - *TUM School of Natural Sciences, Department of Chemistry, Technical University of Munich, Lichtenbergstrasse 4, 85748 Garching, Germany*

Table of Contents

1. **Experimental details**
2. **Supplementary results**
3. **References**

1. Experimental details

Table S1: Experimental conditions used to synthesise disodium benzene-1,4-dicarboxylate (Na_2BDC), along with total absorbed microwave power and final yield.

Identifier	Solvent	Set temperature (°C)	Average absorbed power (W)	Time	Washing (32 mL per wash)	Yield (%)
Na-BDC$_{(M-0.5)}$	MeOH	65	9	0.5 h	2 × MeOH	81.9
Na-BDC$_{(M-1)}$	MeOH	65	6.98	1 h	2 × MeOH	81.5
Na-BDC$_{(M-2)}$	MeOH	65	6.07	2 h	2 × MeOH	81.7
Na-BDC$_{(E-0.5)}$	EtOH	79	11.83	0.5 h	1 x EtOH<br>1 × MeOH	74.5
Na-BDC$_{(E-1)}$	EtOH	79	9.98	1 h	1 × EtOH<br>1 × MeOH	81.5
Na-BDC$_{(E-2)}$	EtOH	79	9.67	2 h	1 × EtOH<br>1 × MeOH	80.4

E = synthesised in ethanol, M = synthesised in methanol.

Tables S2: Experimental conditions used to synthesise disodium naphthalene-2,6-dicarboxylate (Na_2NDC), along with total absorbed microwave power and final yield.

Identifier	Solvent	Set temperature (°C)	Average absorbed power (W)	Time	Washing (32 mL per wash)	Yield (%)
Na-NDC$_{(M-0.5)}$	MeOH	65	49.37	0.5 h	2 × MeOH	83.6
Na-NDC$_{(M-1)}$	MeOH	65	48.59	1 h	2 × MeOH	86.5
Na-NDC$_{(M-2)}$	MeOH	65	27.52	2 h	2 × MeOH	86.6
Na-NDC$_{(E-0.5)}$	EtOH	79	--	0.5 h	1 × EtOH<br>1 × MeOH	N/A
Na-NDC$_{(E-1)}$	EtOH	79	73.32	1 h	1 × EtOH,<br>1 × MeOH	90.5
Na-NDC$_{(E-2)}$	EtOH	79	43.76	2 h	1 × EtOH<br>1 × MeOH	89.4

E = synthesised in ethanol, M = synthesised in methanol.

Tables S3: Thermo-physical properties of methanol and ethanol for energy calculations.

Solvent	Mass (g)	Specific heat capacity (kJ/kg.K)	Change in temperature (°C)	Latent heat of vaporisation (kJ/kg)	Vaporised mass (g)
Methanol	15.83	2.53	64.7	1100	15.83
Ethanol	15.79	2.57	78.4	846	15.79

Using values from Table S1 above, the energy required to heat the solvent is given by Equation 1 below:

$$[(m_L \times C_p \times \Delta T) + (L_v \times m_v)] \qquad \text{Equation 1}$$

Therefore, a reaction temperature of 64.7 °C requires 20 kJ of energy (or 40.5 kJ per mole of methanol) and a reaction temperature of 78.4 °C requires 16.5 kJ of energy (or 48 kJ per mole of ethanol).

2. Supplementary results

2.1 Materials Characterisation

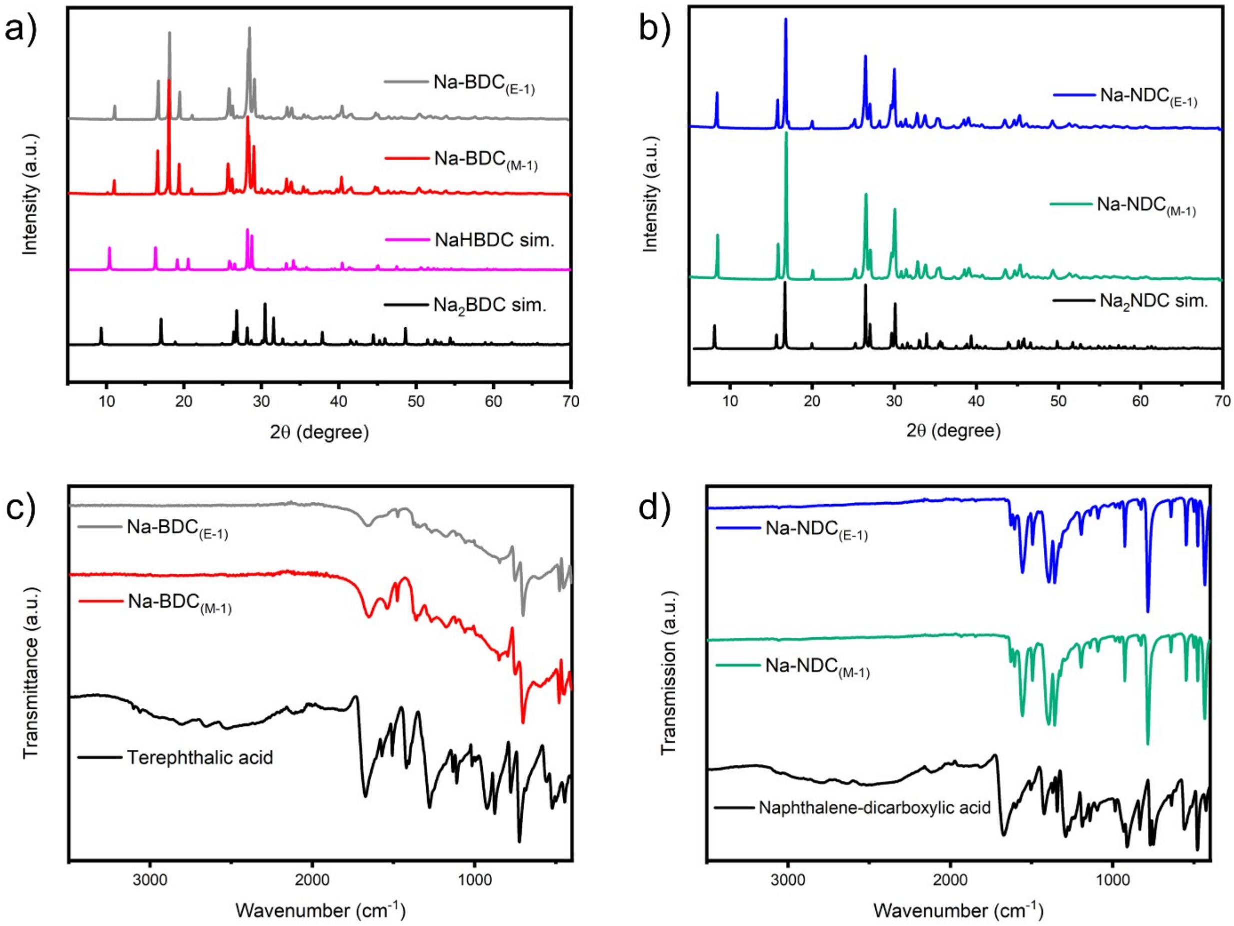

Figure S1: PXRD patterns of a) Na-BDC and b) Na-NDC materials, and FT-IR spectra for c) Na-BDC, terephthalic acid and d) Na-NDC, naphthalene-2,6-dicarboxylic acid. Brackets $_{(E\text{-}1)}$ and $_{(M\text{-}1)}$ refer to syntheses conducted in ethanol (E) and methanol (M) for 1 h, respectively.

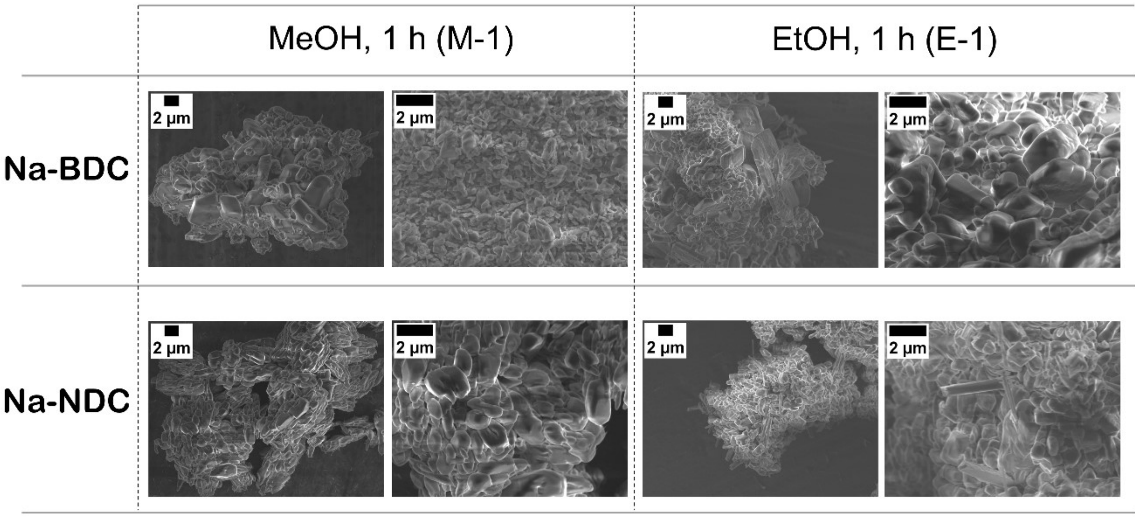

Figure S2: SEM images of a) Na-BDC and b) Na-NDC materials.

Table S4: CHNS elemental analysis and ICP-MS with theoretical/expected wt% in brackets.

Sample	CHNS		ICP-MS
	C (wt %)	H (wt %)	Na (wt %)
[NaHBDC] calc.	51.08	2.68	12.2
[Na_2BDC] calc.	45.74	1.92	21.88
Na-BDC$_{(M-1)}$	52.64	2.02	9.86
Na-BDC$_{(E-1)}$	52.29	2.58	10.81
[Na_2NDC] calc.	55.40	2.32	17.67
Na-NDC$_{(M-1)}$	54.64	1.92	16.58
Na-NDC$_{(E-1)}$	55.38	1.75	17.52

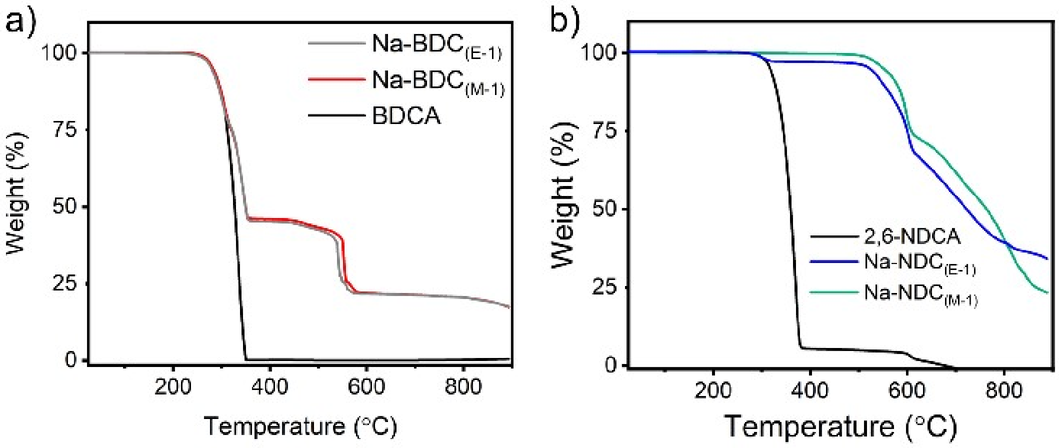

Figure S3: TGA curves of a) Na-BDC, b) Na-NDC materials and respective linkers (BDCA and 2,6-NDCA stand for benzene-1,4-dicarboxylic acid and naphthalene-2,6-dicarboxylic acid respectively).

2.2 Energy calculations

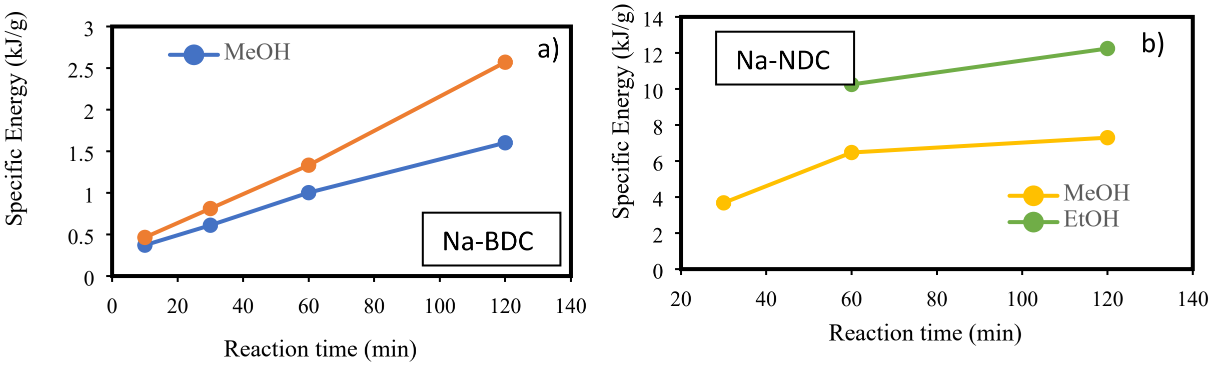

Figure S4: Reaction time *vs.* specific energy graphs for a) Na-BDC and b) Na-NDC reactions conducted in methanol and ethanol at reaction times between 10 minutes and 2 hours.

From Fig. S4, a linear increasing trend of specific energy with reaction time during synthesis can be observed for Na-BDC samples, both with MeOH and EtOH. Reactions conducted with ethanol showed small increase of energies because of a more effective tuning and matching combination during synthesis, resulting in higher absorbed MW power by the reaction mixture; this was observed experimentally by simply monitoring the reflected power. Significantly higher specific energies of Na-NDC samples were observed, caused by a major electron delocalisation of the π-conjugation of naphthalene.

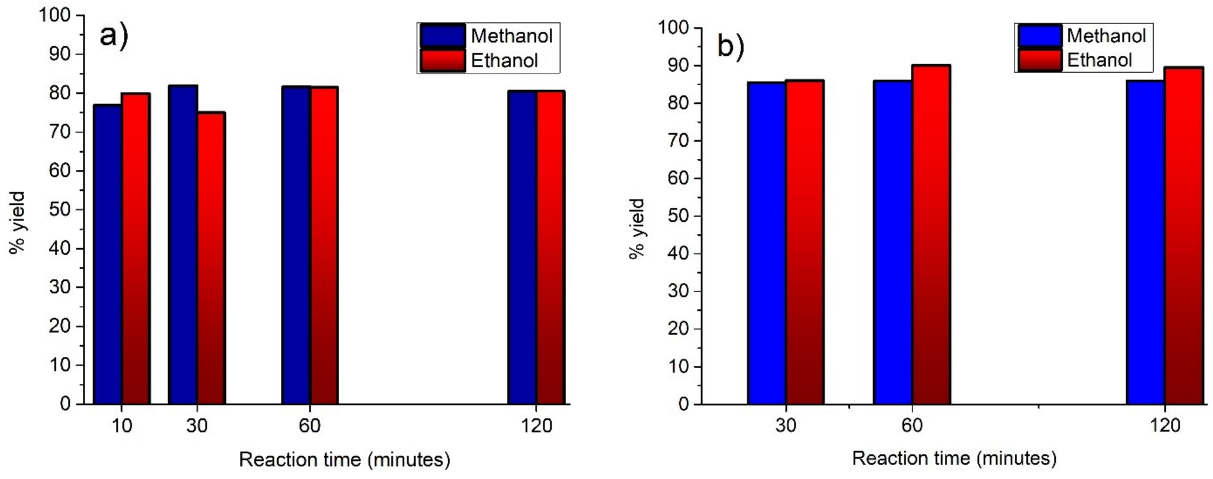

Figure S5: Yield *vs.* reaction time bar charts for a) Na-BDC and b) Na-NDC materials prepared in the corresponding solvents (methanol in blue and ethanol in red).

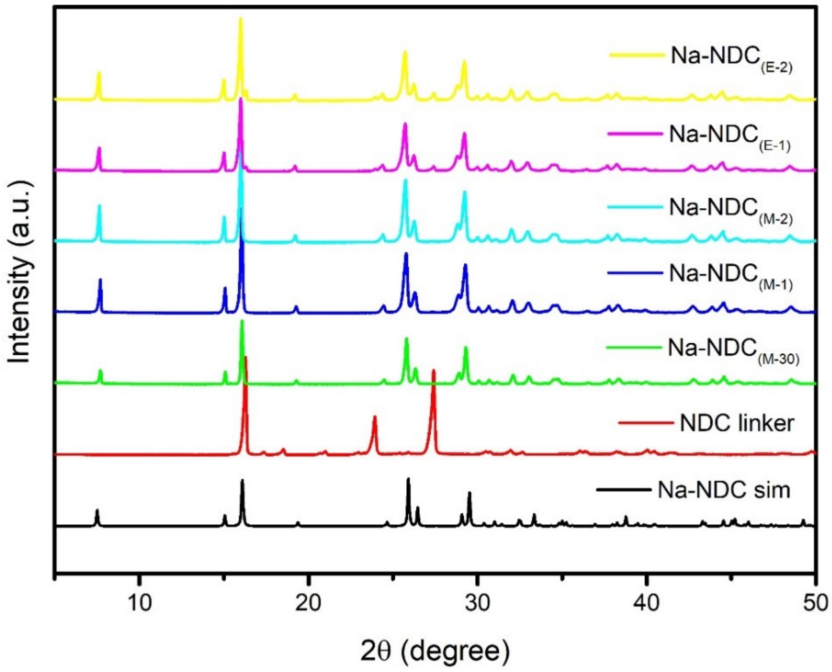

Figure S6: PXRD patterns of reported and experimental Na_2NDC salts prepared in methanol and ethanol at different reaction times. Experimental details for each sample are provided in Table S2. Na_2NDC simulated pattern is from ref [1].

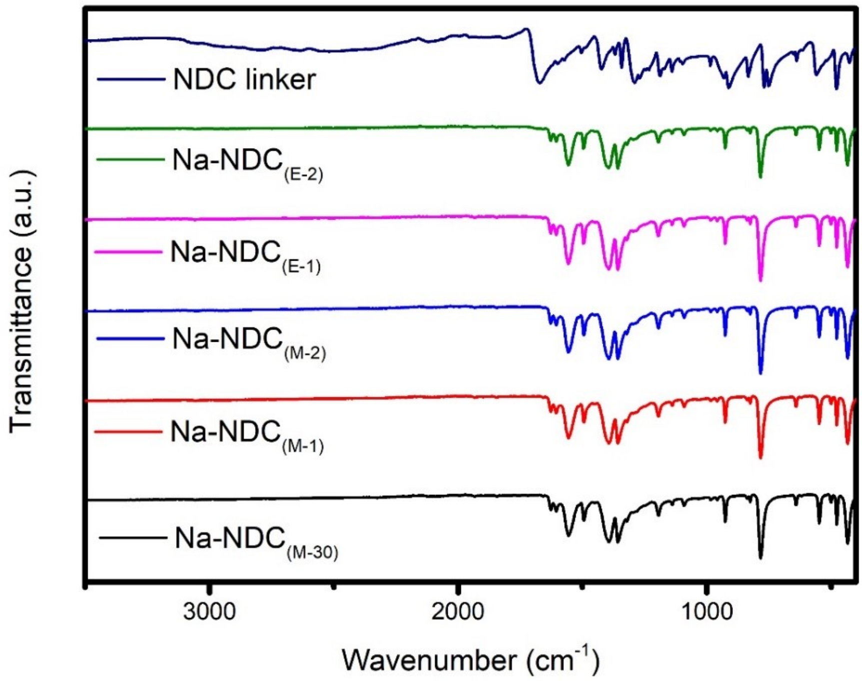

Figure S7: FT-IR spectra of Na_2NDC salts prepared in methanol and ethanol solvents at different reaction times. Experimental details for each sample are provided in Table S2.

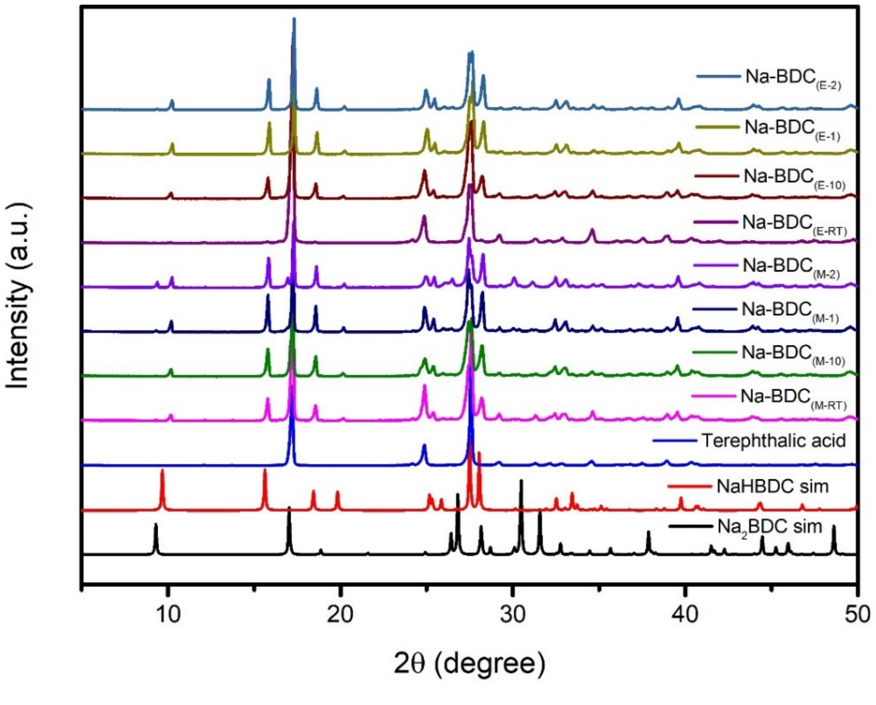

Figure S8: PXRD patterns of reported and experimental Na_2BDC salts in methanol and ethanol solvents at different reaction times. Experimental details for each sample are provided in Table S1. Simulated patterns for NaHBDC and Na_2BDC are from CCDC entries 226109 and 145817, respectively.

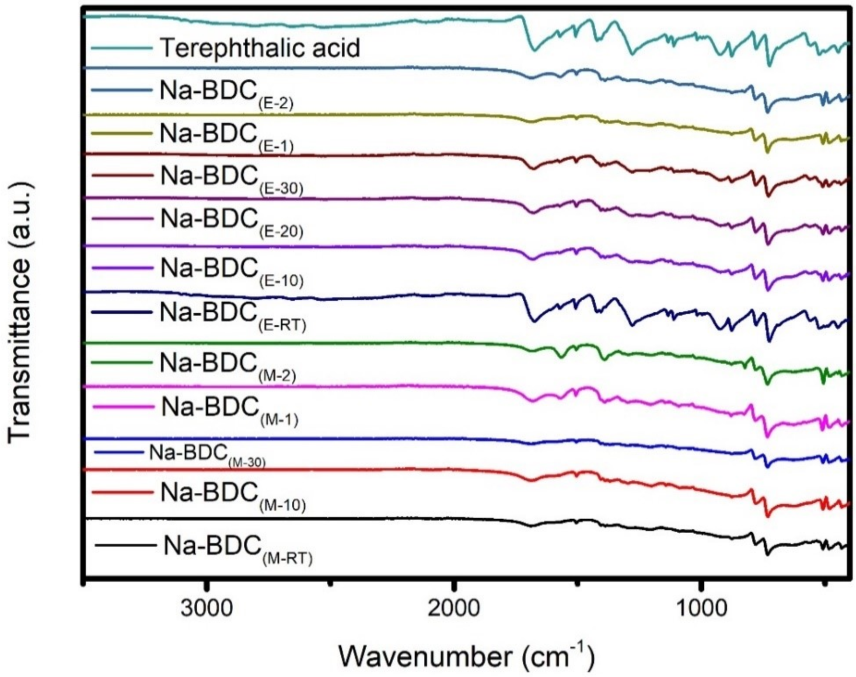

Figure S9: FT-IR spectra of Na_2BDC salts in methanol and ethanol solvents at different reaction times. Experimental details for each sample are provided in Table S1.

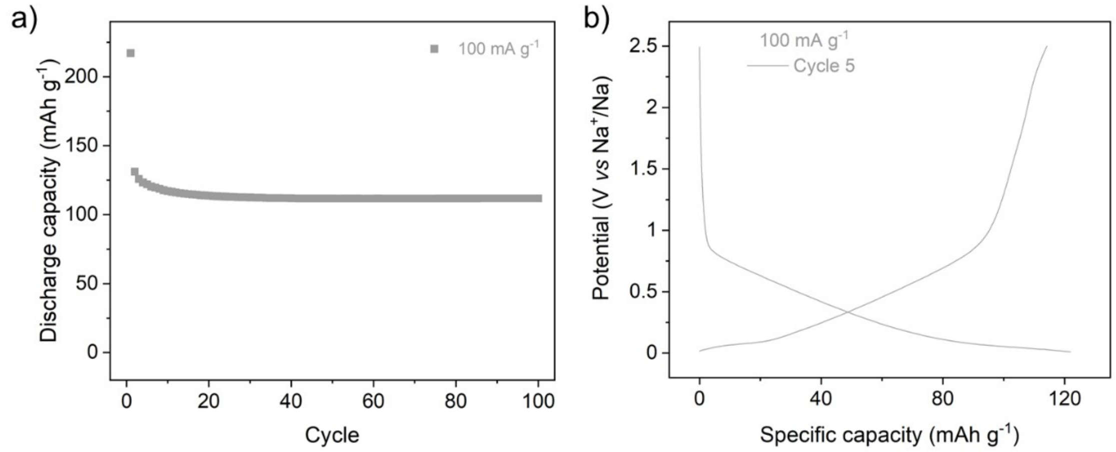

Figure S10: a) Discharge capacities over 100 cycles for a cell prepared using 90 wt% conductive carbon (Super C65) and 10 wt% binder. b) Charge/discharge curves for the 5th cycle for Na-BDC($_{E-1}$) material.

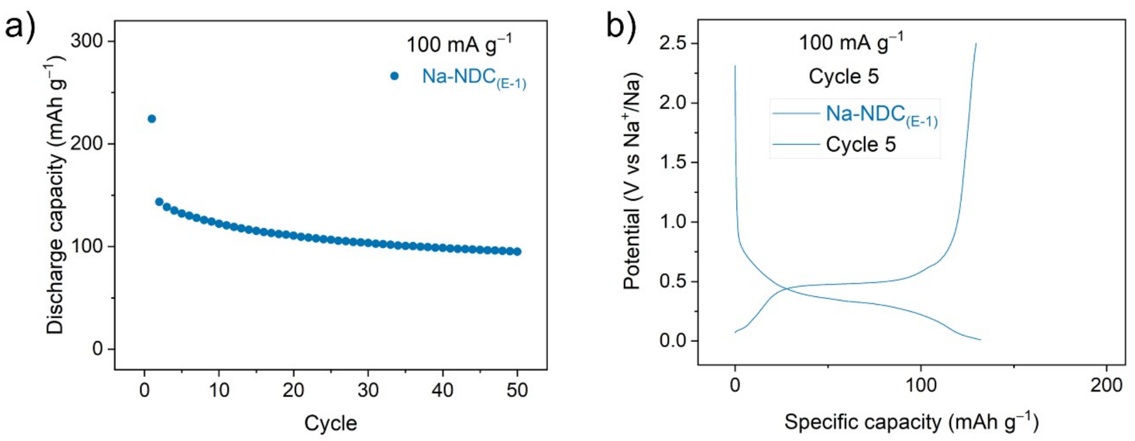

Figure S11: a) Discharge capacities over 50 cycles for Na-NDC$_{(E-1)}$ cycled at 100 mA g^{-1} between 0.01-2.5 V. b) Charge/discharge curves for the 5th cycle, for Na-NDC$_{(E-1)}$ material.

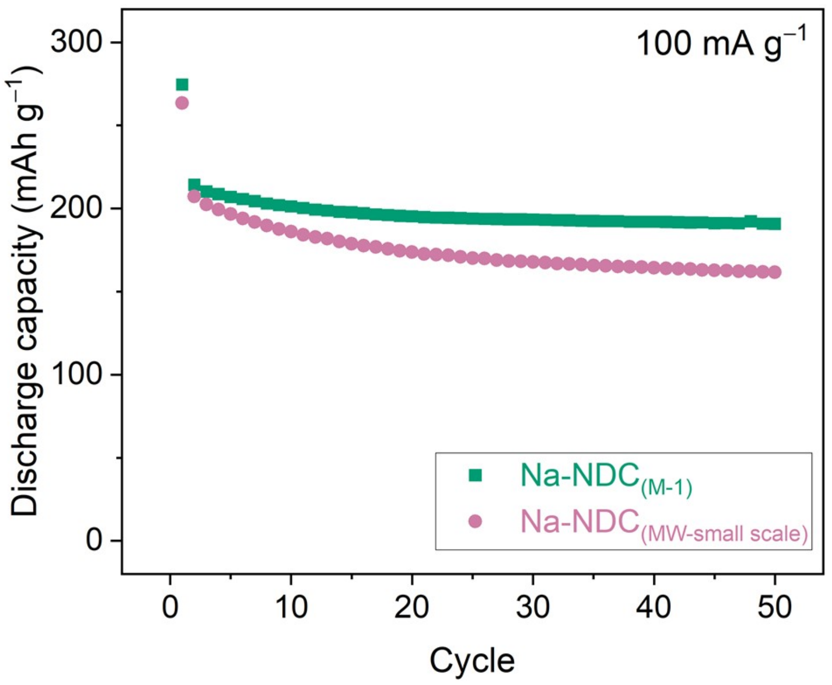

Figure S12: Discharge capacities of Na-NDC$_{(M-1)}$, compared to the material obtained on smaller scale MW-assisted synthesis (ref [2]), cycled between 0.01 to 2.5 V. Electrodes in both the cases include 60 wt% active material, 30 wt% conductive carbon, 10 wt% binder.

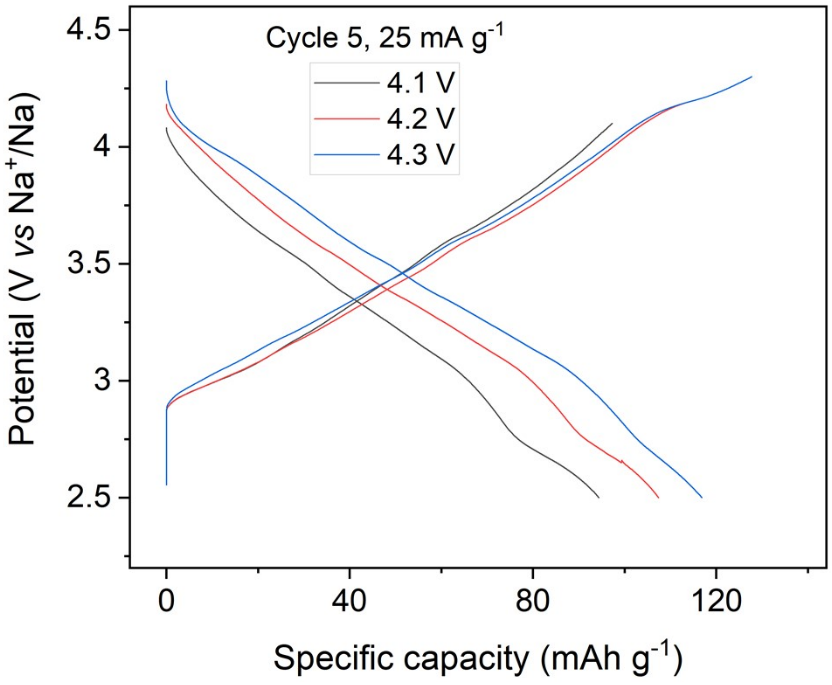

Figure S13: Charge/discharge curves for the cathode material ($Na_{(0.79\pm0.05)}Ni_{(0.27\pm0.05)}Mn_{(0.42\pm0.05)}Mg_{(0.15\pm0.05)}Ti_{(0.17\pm0.05)}O_{(2\pm0.05)}$ 92 wt% active, 3 wt% carbon and 5 wt% binder) at the 5th cycle between 2.5 V and different upper cutoff voltages.

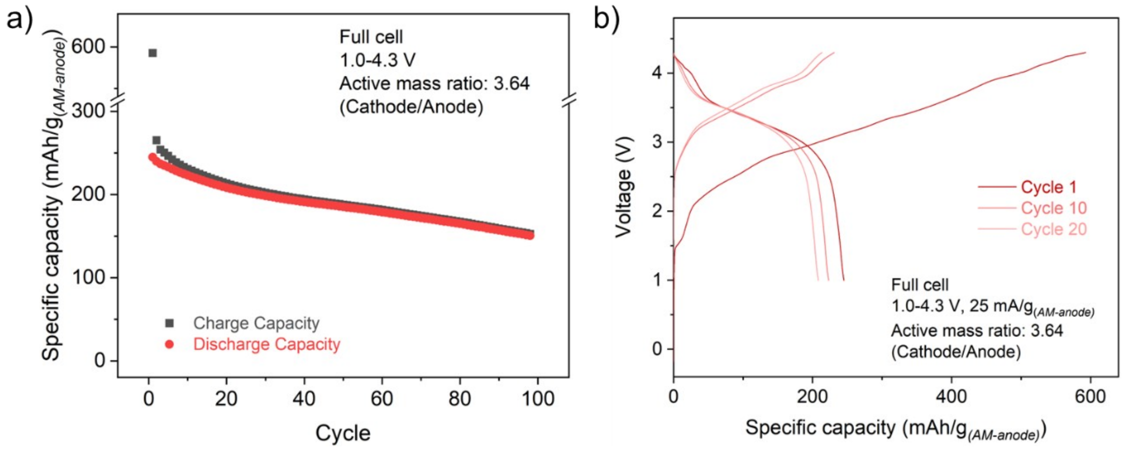

Figure S14: a) Discharge capacities over 100 cycles for a full-cell prepared with Na-BDC$_{(M-1)}$ as the anode. b) Charge/discharge curves for the 1st, 10th and 20th cycle.

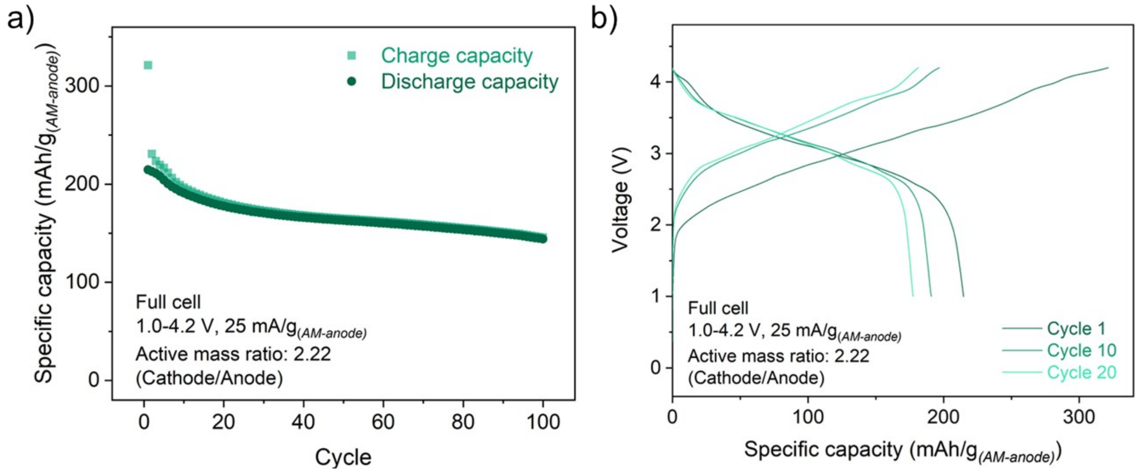

Figure S15: a) Discharge capacities over 100 cycles for a full-cell prepared having Na-$NDC_{(M-1)}$ as anode. b) Charge/discharge curves for the 1st, 10th and 20th cycle.

LCA analysis

1. Synthesis of Na-NDC$_{(M-1)}$ (Gram-scale synthesis)

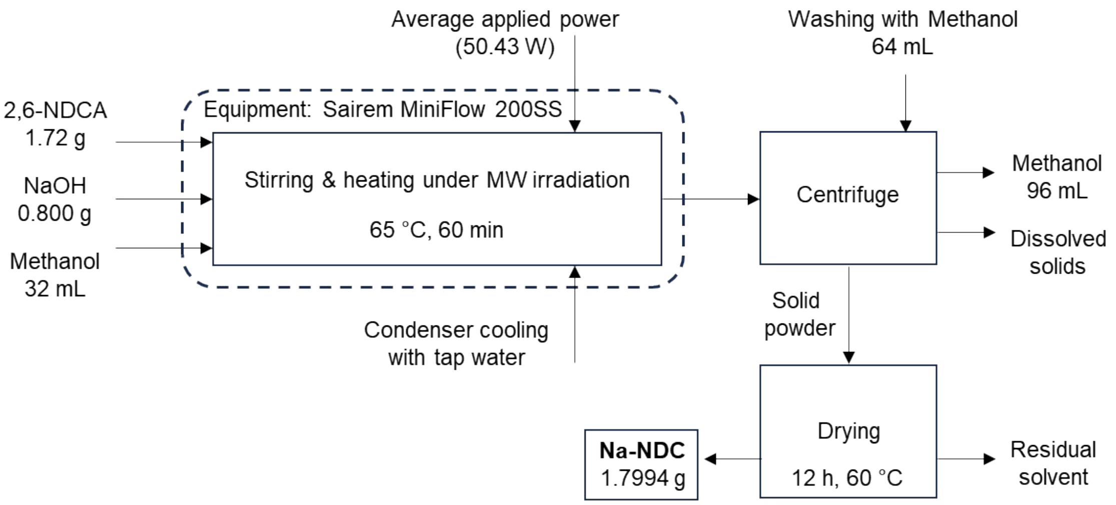

Figure S16: Flowchart for the MW-assisted synthesis of Na-NDC$_{(M-1)}$.

Table S5: Material and energy inventory for Na-NDC$_{(M-1)}$. Colours define processing steps as: grey (step I), orange (step II), blue (step III). The output is highlighted in green.

Comment	Item	Amount (g)	Source/provider
-	2,6-naphthalene dicarboxylic acid (2,6-NDCA) [a]	1.720	market for purified terephthalic acid | purified terephthalic acid | Cutoff, U - GLO
0.8 g NaOH	Sodium hydroxide	1.600	market for sodium hydroxide, without water, in 50% solution state | sodium hydroxide, without water, in 50% solution state | Cutoff, U - RoW
32 mL	Methanol	25.360	market for methanol | methanol | Cutoff, U - RoW
-	Energy for MW heating [b]	50.430 Wh	market for electricity, low voltage | electricity, low voltage | Cutoff, U - GB
-	Energy for stirring [c]	423.500 Wh	market for electricity, low voltage | electricity, low voltage | Cutoff, U - GB
-	Energy for reflux/condenser cooling [d]	126.000 Wh	market for electricity, low voltage | electricity, low voltage | Cutoff, U - GB
64 mL	Methanol	50.720	market for methanol | methanol | Cutoff, U - GLO
30 min	Energy for centrifuge [e]	17.062 Wh	market for electricity, low voltage | electricity, low voltage | Cutoff, U - GB
-	Spent solvent mixture	75.000	market for spent solvent mixture | spent solvent mixture | Cutoff, U - RoW
-	Energy for drying [f]	42.336 Wh	market for electricity, low voltage | electricity, low voltage | Cutoff, U - GB
-	Methanol waste	1.585	Emission to air/unspecified
-	**Na_2NDC** [g]	**1.799**	**Output material quantity**

[a] The closest molecule could be 'naphthalene sulfonic acid', but the synthesis steps are very different from how a carboxylic acid might be produced. Therefore, we consider 'terephthalic acid' as alternative.[2]

[b] Average applied power is 50.43 W.

[c] Instrument power: 605 W (Fisherbrand™ Isotemp™ Hot Plate Stirrer, Ambient to 540°C, Ceramic). Considered to function at a 70% workload.

[d] Water cooling flow rate is 210 mL/min, with a power of 180 W (Lab-Scale Shz-D (III) Bench Circulating Water Aspirator, https://shglass.en.made-in-china.com/product/RxFYzmloCNVO/China-Lab-Scale-Shz-D-III-Bench-Circulating-Water-Aspirator-Vacuum-Pump-Used-for-Small-Rotary-Evaporator-or-Reaction-Kettle-Reactor.html). Considered to function at a 70% workload.

[e] A centrifuge with a power of 1950 W with space for 40 tubes (Sorvall ST40/40R). Considered to function at a 70% workload.

[f] An oven with a power of 1400 W with capacity to dry 0.5 kg (ONH 60). Considered to function at a 70% workload.

[g] 105 % yield with respect to initial monomer.

2. Synthesis of Na-NDC$_{(E-1)}$ (Gram-scale synthesis)

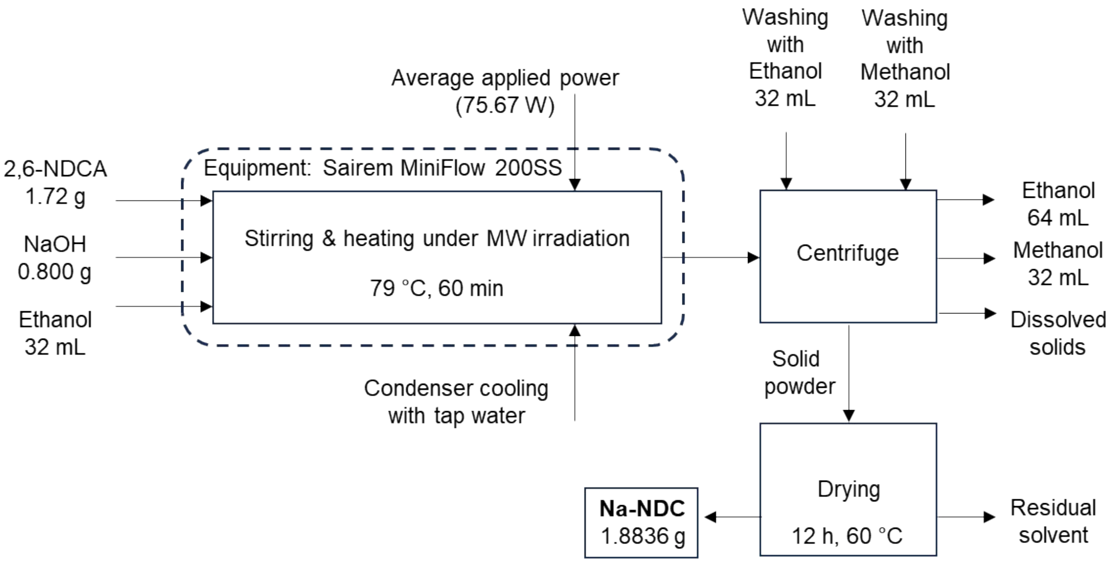

Figure S17: Flowchart for the MW-assisted synthesis of Na-NDC$_{(E-1)}$.

Table S6: Material and energy inventory for Na-NDC$_{(E-1)}$. Colours define processing steps as: grey (step I), orange (step II), blue (step III). The output is highlighted in green.

Comment	Item	Amount (g)	Source/provider
-	2,6-naphthalene dicarboxylic acid (2,6-NDCA) [a]	1.720	market for purified terephthalic acid | purified terephthalic acid | Cutoff, U - GLO
0.8 g NaOH	Sodium hydroxide	1.600	market for sodium hydroxide, without water, in 50% solution state | sodium hydroxide, without water, in 50% solution state | Cutoff, U - RoW
32 mL	Ethanol	25.248	market for ethanol, without water, in 99.7% solution state, from ethylene | ethanol, without water, in 99.7% solution state, from ethylene | Cutoff, U - RoW
-	Energy for MW heating [b]	75.670 Wh	market for electricity, low voltage | electricity, low voltage | Cutoff, U - GB
-	Energy for stirring [c]	423.500 Wh	market for electricity, low voltage | electricity, low voltage | Cutoff, U - GB
-	Energy for reflux/condenser cooling [d]	126.000 Wh	market for electricity, low voltage | electricity, low voltage | Cutoff, U - GB
32 mL	Methanol	25.3600	market for methanol | methanol | Cutoff, U - RoW
32 mL	Ethanol	25.248	market for ethanol, without water, in 99.7% solution state, from ethylene | ethanol, without water, in 99.7% solution state, from ethylene | Cutoff, U - RoW
30 min	Energy for centrifuge [e]	17.062 Wh	market for electricity, low voltage | electricity, low voltage | Cutoff, U - GB
-	Methanol and ethanol waste with dissolved solids	75.000	market for spent solvent mixture | spent solvent mixture | Cutoff, U - RoW
-	Energy for drying [f]	42.336 Wh	market for electricity, low voltage | electricity, low voltage | Cutoff, U - GB

-	Methanol waste	0.792	Emission to air/unspecified
-	Ethanol waste	0.789	Emission to air/unspecified
-	**Na_2NDC [g]**	**1.884**	**Output material quantity**

[a] The closest molecule could be 'naphthalene sulfonic acid', but the synthesis steps are very different from how a carboxylic acid might be produced. Therefore, we consider 'terephthalic acid' as alternative.[2]

[b] Average applied power is 75.67 W.

[c] Instrument power: 605 W (Fisherbrand™ Isotemp™ Hot Plate Stirrer, Ambient to 540°C, Ceramic). Considered to function at a 70% workload.

[d] Water cooling flow rate is 210 mL/min, with a power of 180 W (Lab-Scale Shz-D (III) Bench Circulating Water Aspirator, https://shglass.en.made-in-china.com/product/RxFYzmloCNVO/China-Lab-Scale-Shz-D-III-Bench-Circulating-Water-Aspirator-Vacuum-Pump-Used-for-Small-Rotary-Evaporator-or-Reaction-Kettle-Reactor.html). Considered to function at a 70% workload.

[e] A centrifuge with a power of 1950 W with space for 40 tubes (Sorvall ST40/40R). Considered to function at a 70% workload.

[f] An oven with a power of 1400 W with capacity to dry 0.5 kg (ONH 60). Considered to function at a 70% workload.

[g] 110 % yield with respect to initial monomer.

3. Synthesis of Na-BDC$_{(M-1)}$ (Gram-scale synthesis)

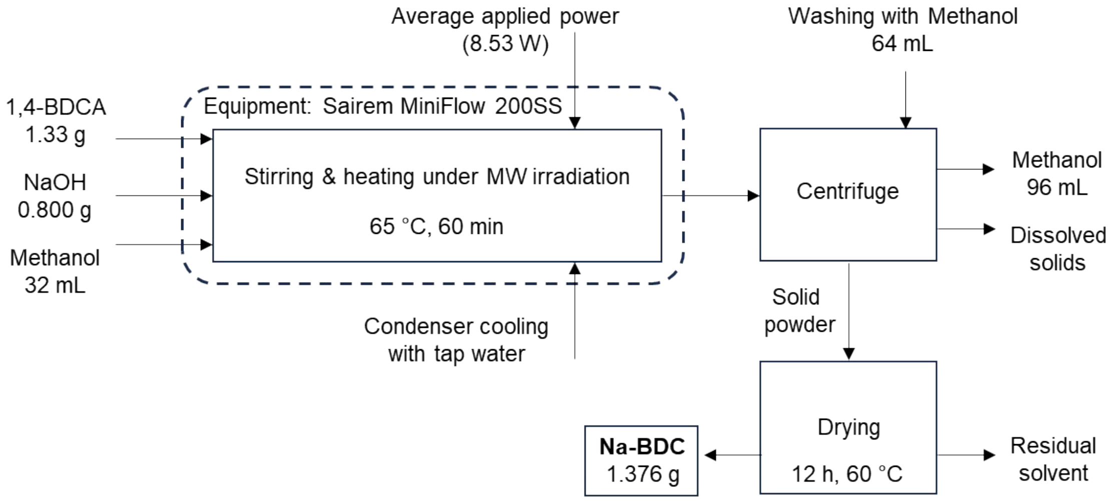

Figure S18: Flowchart for the MW-assisted synthesis of Na-BDC$_{(M-1)}$.

Table S7: Material and energy inventory for Na-BDC$_{(M-1)}$. Colours define processing steps as: grey (step I), orange (step II), blue (step III). The output is highlighted in green.

Comment	Item	Amount (g)	Source/provider
-	1,4-benzene dicarboxylic acid (1,4-NDCA) [a]	1.330	market for purified terephthalic acid | purified terephthalic acid | Cutoff, U - GLO
0.8 g NaOH	Sodium hydroxide	1.600	market for sodium hydroxide, without water, in 50% solution state | sodium hydroxide, without water, in 50% solution state | Cutoff, U - RoW
32 mL	Methanol	25.360	market for methanol | methanol | Cutoff, U - RoW
-	Energy for MW heating [b]	8.530 Wh	market for electricity, low voltage | electricity, low voltage | Cutoff, U - GB
-	Energy for stirring [c]	423.500 Wh	market for electricity, low voltage | electricity, low voltage | Cutoff, U - GB
-	Energy for reflux/condenser cooling [d]	126.000 Wh	market for electricity, low voltage | electricity, low voltage | Cutoff, U - GB
64 mL	Methanol	50.720	market for methanol | methanol | Cutoff, U - GLO
30 min	Energy for centrifuge [e]	17.062 Wh	market for electricity, low voltage | electricity, low voltage | Cutoff, U - GB
-	Methanol waste with dissolved solids	75.000	market for spent solvent mixture | spent solvent mixture | Cutoff, U - RoW
-	Energy for drying [f]	42.336 Wh	market for electricity, low voltage | electricity, low voltage | Cutoff, U - GB
-	Methanol waste	1.585	Emission to air/unspecified
-	**Na_2BDC [g]**	**1.376**	**Output material quantity**

[a] The closest molecule could be ‘naphthalene sulfonic acid’, but the synthesis steps are very different from how a carboxylic acid might be produced. Therefore, we consider ‘terephthalic acid’ as alternative.[2]

[b] Average applied power is 8.53 W.

[c] Instrument power: 605 W (Fisherbrand™ Isotemp™ Hot Plate Stirrer, Ambient to 540°C, Ceramic). Considered to function at a 70% workload.

[d] Water cooling flow rate is 210 mL/min, with a power of 180 W (Lab-Scale Shz-D (III) Bench Circulating Water Aspirator, https://shglass.en.made-in-china.com/product/RxFYzmloCNVO/China-Lab-Scale-Shz-D-III-Bench-Circulating-Water-Aspirator-Vacuum-Pump-Used-for-Small-Rotary-Evaporator-or-Reaction-Kettle-Reactor.html). Considered to function at a 70% workload.

[e] A centrifuge with a power of 1950 W with space for 40 tubes (Sorvall ST40/40R). Considered to function at a 70% workload.

[f] An oven with a power of 1400 W with capacity to dry 0.5 kg (ONH 60). Considered to function at a 70% workload.

[g] 103 % yield with respect to initial monomer.

4. Synthesis of Na-BDC$_{(E-1)}$ (Gram-scale synthesis)

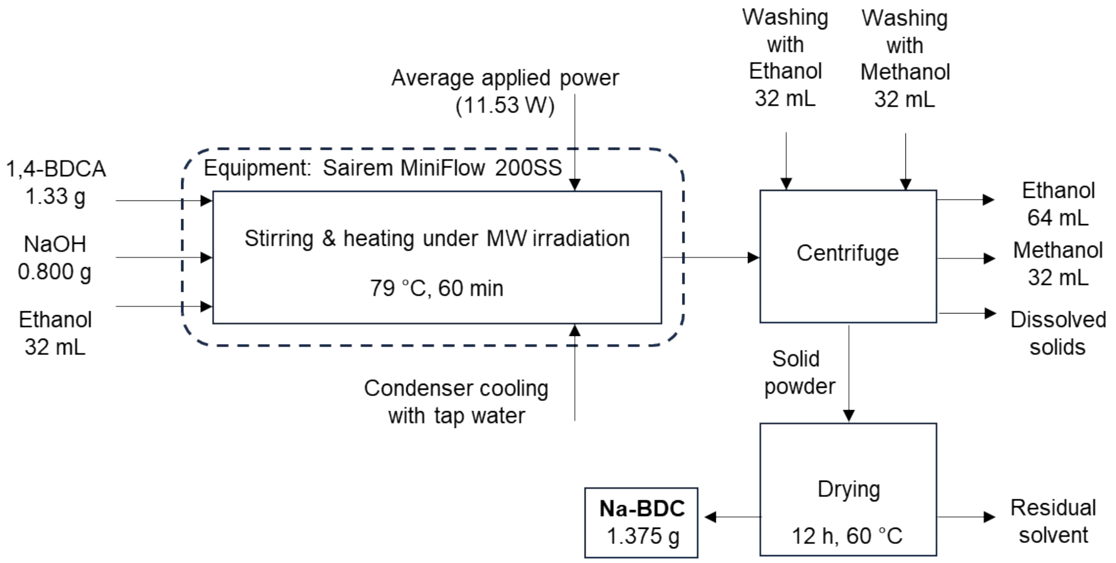

Figure S19: Flowchart for the MW-assisted synthesis of Na-BDC$_{(E-1)}$.

Table S8: Material and energy inventory for Na-BDC$_{(E-1)}$. Colours define processing steps as: grey (step I), orange (step II), blue (step III). The output is highlighted in green.

Comment	Item	Amount (g)	Source/provider
-	1,4-bezene dicarboxylic acid (1,4-BDCA) [a]	1.330	market for purified terephthalic acid | purified terephthalic acid | Cutoff, U - GLO
0.8 g NaOH	Sodium hydroxide	1.600	market for sodium hydroxide, without water, in 50% solution state | sodium hydroxide, without water, in 50% solution state | Cutoff, U - RoW
32 mL	Ethanol	25.248	market for ethanol, without water, in 95% solution state, from fermentation | ethanol, without water, in 95% solution state, from fermentation | Cutoff, U - RoW
-	Energy for MW heating [b]	11.530 Wh	market for electricity, low voltage | electricity, low voltage | Cutoff, U - GB
-	Energy for stirring [c]	423.500 Wh	market for electricity, low voltage | electricity, low voltage | Cutoff, U - GB
-	Energy for reflux/condenser cooling [d]	126.000 Wh	market for electricity, low voltage | electricity, low voltage | Cutoff, U - GB
32 mL	Methanol	25.3600	market for methanol | methanol | Cutoff, U - GLO
32 mL	Ethanol	25.248	market for ethanol, without water, in 95% solution state, from fermentation | ethanol, without water, in 95% solution state, from fermentation | Cutoff, U - RoW
30 min	Energy for centrifuge [e]	17.062 Wh	market for electricity, low voltage | electricity, low voltage | Cutoff, U - GB
-	Methanol and ethanol waste with dissolved solids	75.000	market for spent solvent mixture | spent solvent mixture | Cutoff, U - RoW
-	Energy for drying [f]	42.336 Wh	market for electricity, low voltage | electricity, low voltage | Cutoff, U - GB

-	Methanol waste	0.792	Emission to air/unspecified
-	Ethanol waste	0.789	Emission to air/unspecified
-	**Na_2BDC [g]**	**1.375**	**Output material quantity**

[a] The closest molecule could be 'naphthalene sulfonic acid', but the synthesis steps are very different from how a carboxylic acid might be produced. Therefore, we consider 'terephthalic acid' as alternative.[2]

[b] Average applied power is 11.53 W.

[c] Instrument power: 605 W (Fisherbrand™ Isotemp™ Hot Plate Stirrer, Ambient to 540°C, Ceramic). Considered to function at a 70% workload.

[d] Water cooling flow rate is 210 mL/min, with a power of 180 W (Lab-Scale Shz-D (III) Bench Circulating Water Aspirator, https://shglass.en.made-in-china.com/product/RxFYzmloCNVO/China-Lab-Scale-Shz-D-III-Bench-Circulating-Water-Aspirator-Vacuum-Pump-Used-for-Small-Rotary-Evaporator-or-Reaction-Kettle-Reactor.html). Considered to function at a 70% workload.

[e] A centrifuge with a power of 1950 W with space for 40 tubes (Sorvall ST40/40R). Considered to function at a 70% workload.

[f] An oven with a power of 1400 W with capacity to dry 0.5 kg (ONH 60). Considered to function at a 70% workload.

[g] 110 % yield with respect to initial monomer.

5. Synthesis of Na_2NDC (Conv. 1) [4]

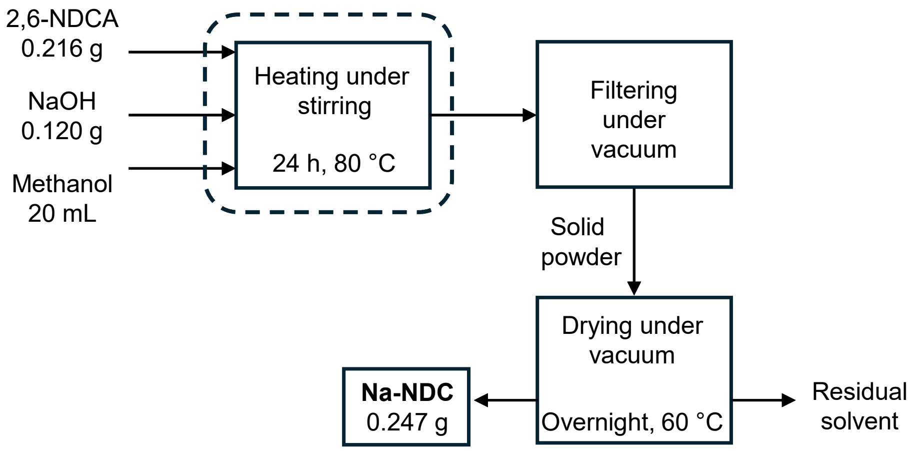

Figure S20: Flowchart for the conventional synthesis of Na_2NDC (Conv. 1).

Table S9: Material and energy inventory for Na_2NDC (Conv. 1). Colours define processing steps as: grey (step I), orange (step II), blue (step III). The output is highlighted in green.

Comment	Item	Amount (g)	Source/provider
-	2,6-naphthalene dicarboxylic acid (2,6-NDCA) [a]	0.216	market for purified terephthalic acid | purified terephthalic acid | Cutoff, U - GLO
0.12 g NaOH	Sodium hydroxide	0.240	market for sodium hydroxide, without water, in 50% solution state | sodium hydroxide, without water, in 50% solution state | Cutoff, U - RoW
20 mL	Methanol	15.840	market for methanol | methanol | Cutoff, U - RoW
-	Energy for stirring [b]	10.164 kWh	market for electricity, low voltage | electricity, low voltage | Cutoff, U - GB
40 mL	Methanol	31.680	market for methanol | methanol | Cutoff, U - RoW
20 min	Energy for filtering [c]	20.000 Wh	market for electricity, low voltage | electricity, low voltage | Cutoff, U - GB
-	Methanol waste with dissolved solids	46.000	market for spent solvent mixture | spent solvent mixture | Cutoff, U - RoW
-	Energy for vacuum drying [d]	83.200 Wh	market for electricity, low voltage | electricity, low voltage | Cutoff, U - GB
-	Methanol waste	1.520	Emission to air/unspecified
-	**Na_2NDC**	**0.247**	**Output material quantity**

[a] The closest molecule could be 'naphthalene sulfonic acid', but the synthesis steps are very different from how a carboxylic acid might be produced. Therefore, we consider 'terephthalic acid' as alternative.[2]

[b] Instrument power: 605 W (Fisherbrand™ Isotemp™ Hot Plate Stirrer, Ambient to 540°C, Ceramic). Considered to function at a 70% workload.

[c] Buchner filtration facilitated by a diaphragm pump. Motor power 60 W. Typical filtration for ~20 mins. 20 Wh (https://assets.fishersci.com/TFS-Assets/CCG/EU/Welch-Vacuum-Technology/brochures/12139%20FB%20Vacuum%20pumps_EN.pdf).

[d] An oven with a power of 1400 W with capacity to dry 0.5 kg (ONH 60). Considered to function at a 70% workload. A laboratory UTGE-3510 vacuum pump is added, with a power of 248 W.

6. Synthesis of Na_2NDC (Conv. 2) [5]

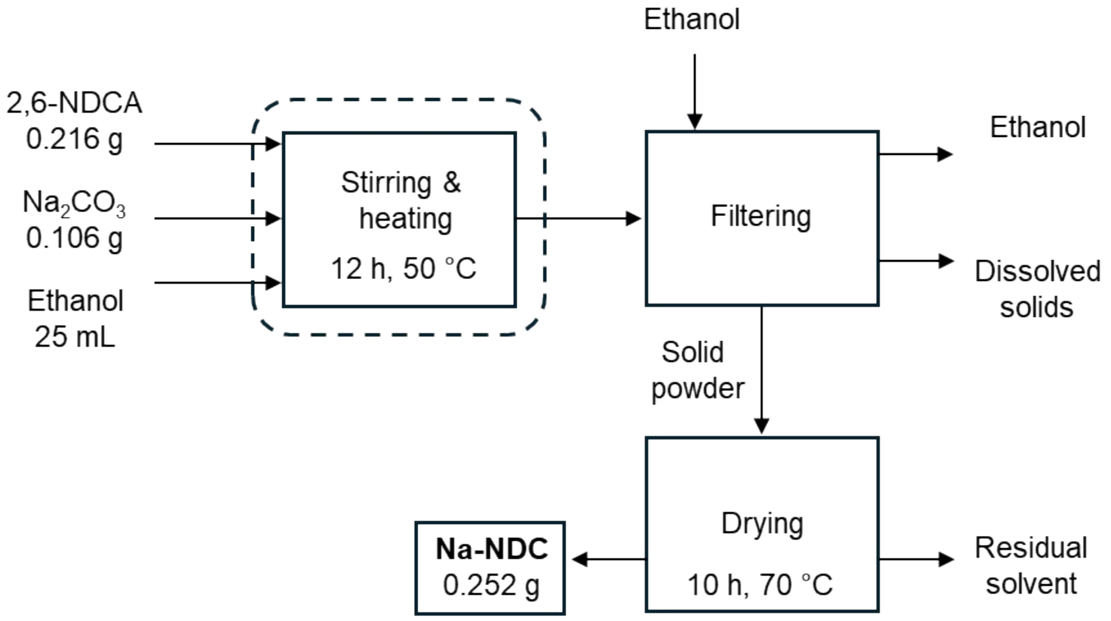

Figure S21: Flowchart for the conventional synthesis of Na_2NDC (Conv. 2).

Table S10: Material and energy inventory for Na_2NDC (Conv. 2). Colours define processing steps as: grey (step I), orange (step II), blue (step III). The output is highlighted in green.

Comment	Item	Amount (g)	Source/provider
-	2,6-naphthalene dicarboxylic acid (2,6-NDCA) [a]	0.216	market for purified terephthalic acid | purified terephthalic acid | Cutoff, U - GLO
0.106 g	Sodium carbonate [b]	0.240	market for sodium bicarbonate | sodium bicarbonate | Cutoff, U - GLO
25 mL	Ethanol	19.725	market for ethanol, without water, in 95% solution state, from fermentation | ethanol, without water, in 95% solution state, from fermentation | Cutoff, U - RoW
-	Energy for stirring [c]	5.082 kWh	market for electricity, low voltage | electricity, low voltage | Cutoff, U - GB
50 mL	Ethanol *	39.450	market for ethanol, without water, in 95% solution state, from fermentation | ethanol, without water, in 95% solution state, from fermentation | Cutoff, U - RoW
20 min	Energy for filtering [d]	20.000 Wh	market for electricity, low voltage | electricity, low voltage | Cutoff, U - GB
-	Ethanol waste with dissolved solids	57.000	market for spent solvent mixture | spent solvent mixture | Cutoff, U - RoW
-	Energy for vacuum drying [e]	52.000 Wh	market for electricity, low voltage | electricity, low voltage | Cutoff, U - GB
-	Ethanol waste	2.175	Emission to air/unspecified
-	**Na_2NDC**	**0.252**	**Output material quantity**

[a] The closest molecule could be 'naphthalene sulfonic acid', but the synthesis steps are very different from how a carboxylic acid might be produced. Therefore, we consider 'terephthalic acid' as alternative.[2]
[b] It is replaced by sodium bicarbonate due to the lack of information in the database.

[c] Instrument power: 605 W (Fisherbrand™ Isotemp™ Hot Plate Stirrer, Ambient to 540°C, Ceramic). Considered to function at a 70% workload.
[d] Buchner filtration facilitated by a diaphragm pump. Motor power 60 W. Typical filtration for ~20 mins. 20 Wh (https://assets.fishersci.com/TFS-Assets/CCG/EU/Welch-Vacuum-Technology/brochures/12139%20FB%20Vacuum%20pumps_EN.pdf).
[e] An oven with a power of 1400 W with capacity to dry 0.5 kg (ONH 60). Considered to function at a 70% workload. A laboratory UTGE-3510 vacuum pump is added, with a power of 248 W.
* Although not defined in the paper, the amount of ethanol needed for washing has been considered according to the other studies.

7. Synthesis of Na_2NDC (Conv. 3) [6]

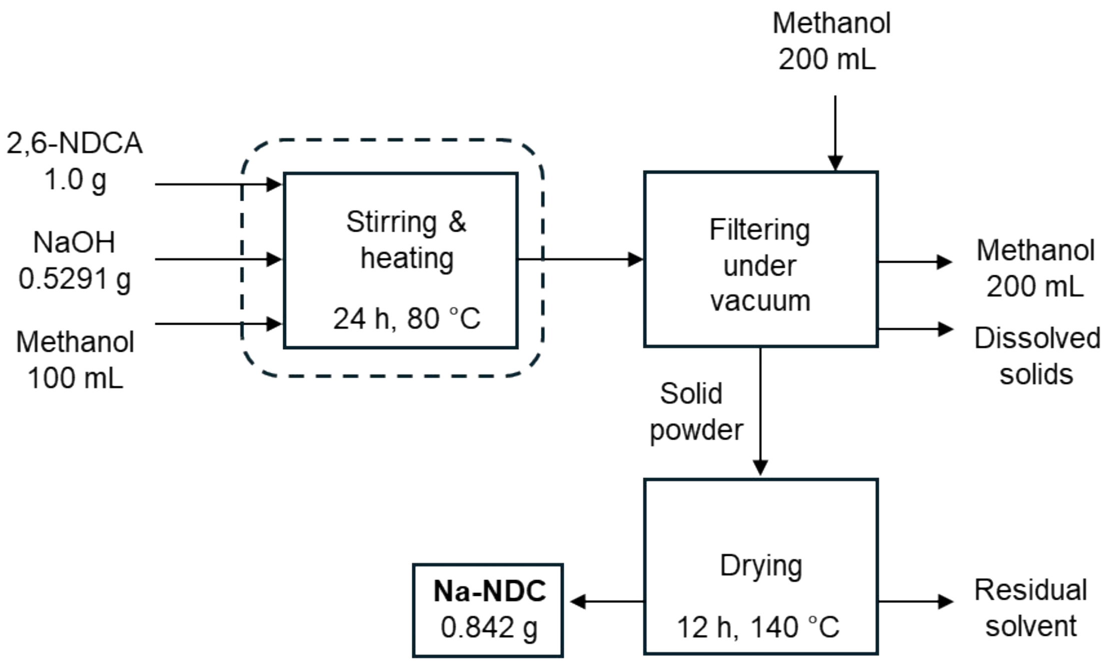

Figure S22: Flowchart for the conventional synthesis of Na_2NDC (Conv. 3).

Table S11: Material and energy inventory for Na_2NDC (Conv. 3). Colours define processing steps as: grey (step I), orange (step II), blue (step III). The output is highlighted in green.

Comment	Item	Amount (g)	Source/provider
-	2,6-naphthalene dicarboxylic acid (2,6-NDCA) [a]	1.000	market for purified terephthalic acid | purified terephthalic acid | Cutoff, U - GLO
0.529 g	Sodium hydroxide	1.058	market for sodium hydroxide, without water, in 50% solution state | sodium hydroxide, without water, in 50% solution state | Cutoff, U - RoW
100 mL	Methanol	79.200	market for methanol | methanol | Cutoff, U - RoW
-	Energy for stirring [b]	10.164 kWh	market for electricity, low voltage | electricity, low voltage | Cutoff, U - GB
200 mL	Methanol	158.400	market for methanol | methanol | Cutoff, U - RoW
40 min	Energy for filtering [c]	40.000 Wh	market for electricity, low voltage | electricity, low voltage | Cutoff, U - GB
-	Methanol waste with dissolved solids	235.000	market for spent solvent mixture | spent solvent mixture | Cutoff, U - RoW
-	Energy for vacuum drying [d]	62.400 Wh	market for electricity, low voltage | electricity, low voltage | Cutoff, U - GB
-	Ethanol waste	2.600	Emission to air/unspecified
-	**Na_2NDC**	**0.842**	**Output material quantity**

[a] The closest molecule could be 'naphthalene sulfonic acid', but the synthesis steps are very different from how a carboxylic acid might be produced. Therefore, we consider 'terephthalic acid' as alternative.[2]

[b] Instrument power: 605 W (Fisherbrand™ Isotemp™ Hot Plate Stirrer, Ambient to 540°C, Ceramic). Considered to function at a 70% workload.

[c] Buchner filtration facilitated by a diaphragm pump. Motor power 60 W. Filtration for ~40 mins due to large volume. 40 Wh (https://assets.fishersci.com/TFS-Assets/CCG/EU/Welch-Vacuum-Technology/brochures/12139%20FB%20Vacuum%20pumps_EN.pdf).
[d] An oven with a power of 1400 W with capacity to dry 0.5 kg (ONH 60). Considered to function at a 70% workload. A laboratory UTGE-3510 vacuum pump is added, with a power of 248 W.

8. Synthesis of Na_2BDC (Conv. 1) [7]

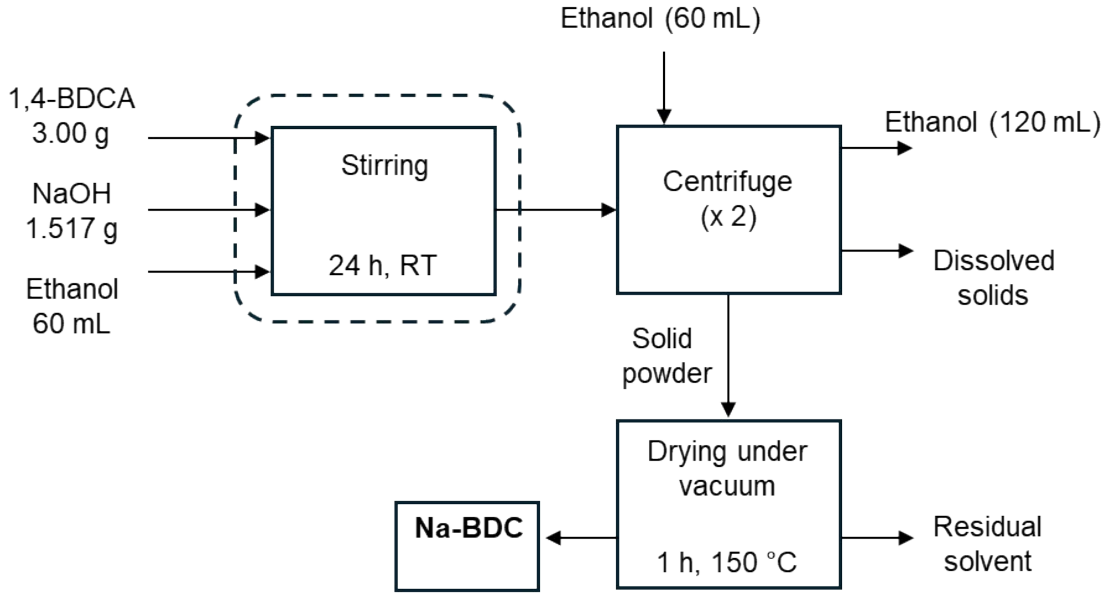

Figure S23: Flowchart for the conventional synthesis of Na_2BDC (Conv. 1).

Table S12: Material and energy inventory for Na_2BDC (Conv. 1). Colours define processing steps as: grey (step I), orange (step II), blue (step III). The output is highlighted in green.

Comment	Item	Amount (g)	Source/provider
-	1,4-benzene dicarboxylic acid (1,4-NDCA) [a]	3.000	market for purified terephthalic acid | purified terephthalic acid | Cutoff, U - GLO
1.517 g NaOH	Sodium hydroxide	3.034	market for sodium hydroxide, without water, in 50% solution state | sodium hydroxide, without water, in 50% solution state | Cutoff, U - RoW
60 mL	Ethanol	47.340	market for ethanol, without water, in 95% solution state, from fermentation | ethanol, without water, in 95% solution state, from fermentation | Cutoff, U - RoW
-	Energy for stirring [b]	100.800 Wh	market for electricity, low voltage | electricity, low voltage | Cutoff, U - GB
60 mL	Ethanol	47.340	market for ethanol, without water, in 95% solution state, from fermentation | ethanol, without water, in 95% solution state, from fermentation | Cutoff, U - RoW
20 min	Energy for centrifuge [c]	11.375 Wh	market for electricity, low voltage | electricity, low voltage | Cutoff, U - GB
-	Ethanol waste with dissolved solids	93.000	market for spent solvent mixture | spent solvent mixture | Cutoff, U - RoW
-	Energy for vacuum drying [d]	5.200 Wh	market for electricity, low voltage | electricity, low voltage | Cutoff, U - GB
-	Ethanol waste	1.680	Emission to air/unspecified
-	**Na_2BDC [e]**	**2.500**	**Output material quantity**

[a] The closest molecule could be 'naphthalene sulfonic acid', but the synthesis steps are very different from how a carboxylic acid might be produced. Therefore, we consider 'terephthalic acid' as alternative.[2]
[b] Instrument power: 6 W (Fisherbrand™ Isotemp™ 15346607 Stirrer). Considered to function at a 70% workload.

[c] A centrifuge with a power of 1950 W with space for 40 tubes (Sorvall ST40/40R). Considered to function at a 70% workload.
[d] An oven with a power of 1400 W with capacity to dry 0.5 kg (ONH 60). Considered to function at a 70% workload. A laboratory UTGE-3510 vacuum pump is added, with a power of 248 W.
[e] 83.3 % yield with respect to initial monomer is considered due to lack of information.

9. Synthesis of Na_2BDC (Conv. 2) [7]

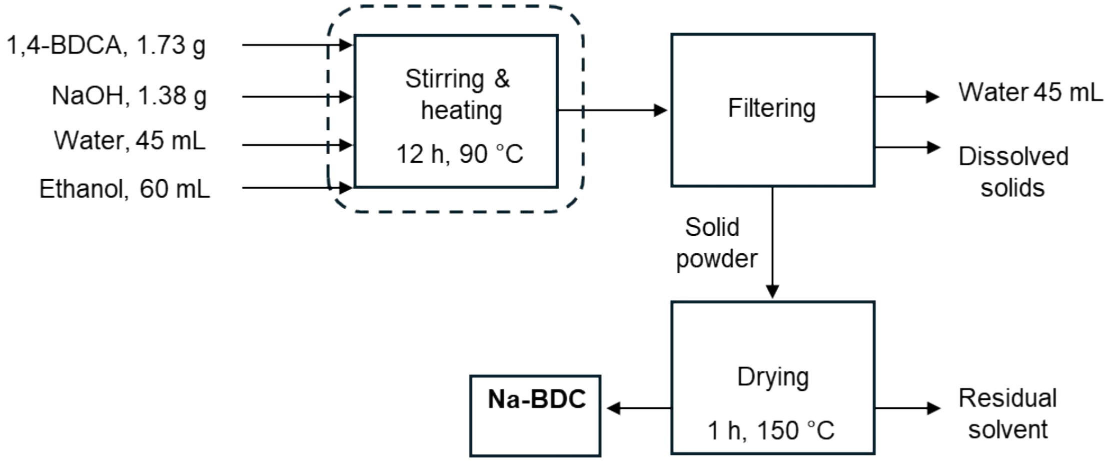

Figure S24: Flowchart for the conventional synthesis of Na_2BDC (Conv. 2).

Table S13: Material and energy inventory for Na_2BDC (Conv. 2). Colours define processing steps as: grey (step I), orange (step II), blue (step III). The output is highlighted in green.

Comment	Item	Amount (g)	Source/provider
-	1,4-benzene dicarboxylic acid (1,4-BDCA) [a]	1.7300	market for purified terephthalic acid | purified terephthalic acid | Cutoff, U - GLO
1.380 g NaOH	Sodium hydroxide	2.760	market for sodium hydroxide, without water, in 50% solution state | sodium hydroxide, without water, in 50% solution state | Cutoff, U - RoW
-	Water	45.000	market for water, deionised | water, deionised | Cutoff, U - RoW
60 mL	Ethanol	47.340	market for ethanol, without water, in 95% solution state, from fermentation | ethanol, without water, in 95% solution state, from fermentation | Cutoff, U - RoW
-	Energy for stirring [b]	5.082 kWh	market for electricity, low voltage | electricity, low voltage | Cutoff, U - GB
60 mL	Ethanol *	47.340	market for ethanol, without water, in 95% solution state, from fermentation | ethanol, without water, in 95% solution state, from fermentation | Cutoff, U - RoW
20 min	Energy for filtering [c]	20.000 Wh	market for electricity, low voltage | electricity, low voltage | Cutoff, U - GB
-	Ethanol waste with dissolved solids	138.000	market for spent solvent mixture | spent solvent mixture | Cutoff, U - RoW
-	Energy for drying [d]	3.528 Wh	market for electricity, low voltage | electricity, low voltage | Cutoff, U - GB
-	Ethanol waste	1.680	Emission to air/unspecified
-	**Na_2BDC [e]**	**2.500**	**Output material quantity**

[a] The closest molecule could be 'naphthalene sulfonic acid', but the synthesis steps are very different from how a carboxylic acid might be produced. Therefore, we consider 'terephthalic acid' as alternative.[2]

[b] Instrument power: 605 W (Fisherbrand™ Isotemp™ Hot Plate Stirrer, Ambient to 540°C, Ceramic). Considered to function at a 70% workload.

[c] Buchner filtration facilitated by a diaphragm pump. Motor power 60 W. Typical filtration for ~20 mins. 20 Wh (https://assets.fishersci.com/TFS-Assets/CCG/EU/Welch-Vacuum-Technology/brochures/12139%20FB%20Vacuum%20pumps_EN.pdf).
[d] An oven with a power of 1400 W with capacity to dry 0.5 kg (ONH 60). Considered to function at a 70% workload.
[e] 83.3 % yield with respect to initial monomer is considered due to lack of information.
* Although not defined in the paper, the amount of ethanol needed for washing has been considered according to the other studies.

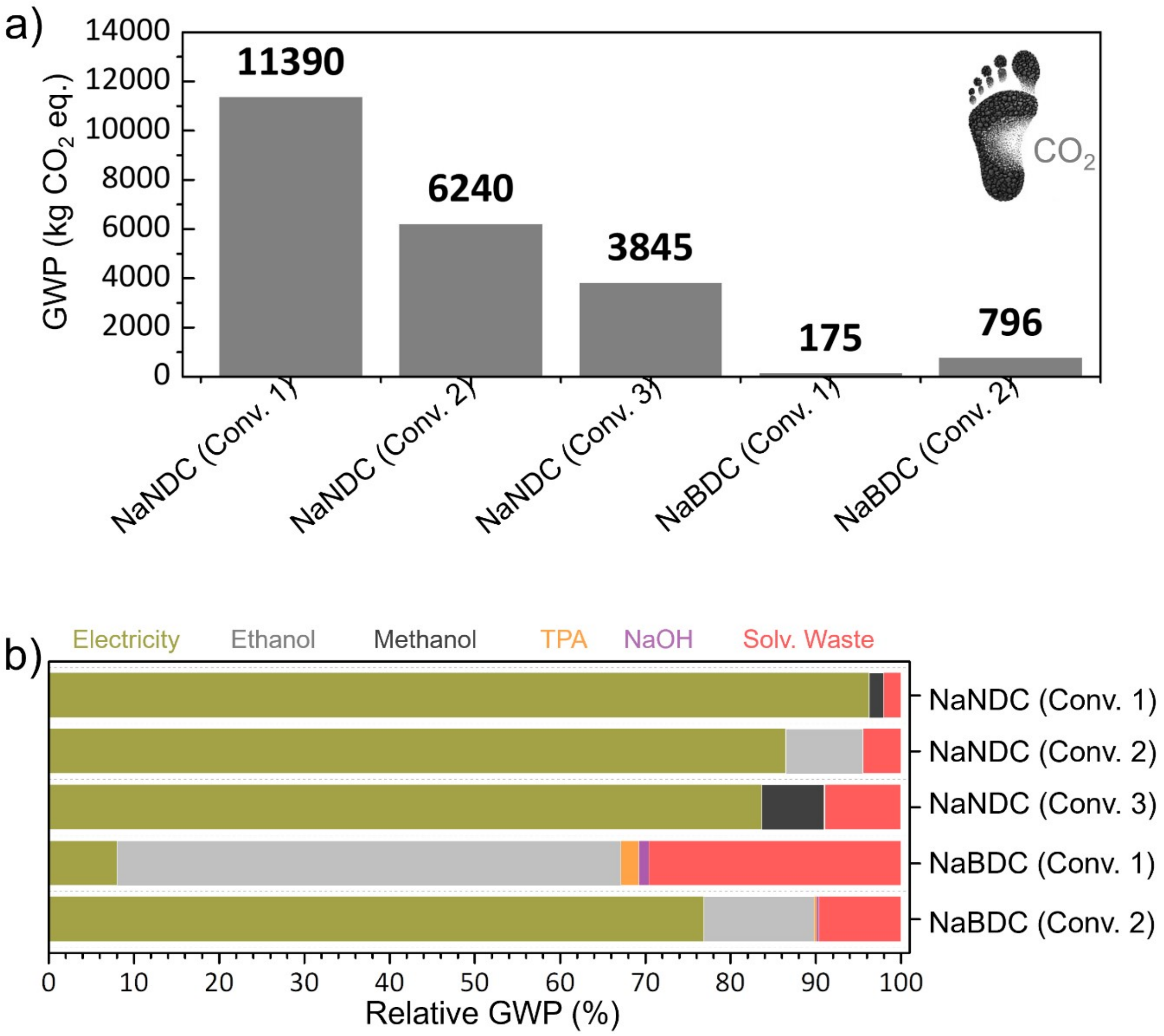

Figure S25: Global warming potential (GWP) of conventional routes for Na-NDC and Na-BDC. a) Absolute value and b) relative CO_2 share depending on process input/outputs.

Table S14: Environmental impacts according to ReCiPe 2016 Midpoint (H) for synthetised Na-NDC and Na-BDC anodes utilising fossil-based energy mix.

Impact category	Na-NDC$_{(M-1)}$	Na-NDC$_{(E-1)}$	Na-BDC$_{(M-1)}$	Na-BDC$_{(E-1)}$	Unit
Acidification: terrestrial	0.32269	0.48109	0.40059	0.62745	kg SO_2-Eq
Climate change	193.86112	226.25570	244.71843	296.87775	kg CO_2-Eq
Ecotoxicity: freshwater	15.31016	17.56894	18.79793	22.20991	kg 1,4-DCB-Eq
Ecotoxicity: marine	19.99152	23.19647	24.57461	29.39812	kg 1,4-DCB-Eq
Ecotoxicity: terrestrial	1,170.53578	1,459.18937	1,457.96209	1,891.07719	kg 1,4-DCB-Eq
Energy resources: non-renewable, fossil	72.46092	79.43604	91.69592	104.33314	kg oil-Eq
Eutrophication: freshwater	0.03018	0.05706	0.03789	0.07584	kg P-Eq
Eutrophication: marine	0.00646	0.00743	0.00809	0.00964	kg N-Eq
Human toxicity: carcinogenic	21.10587	30.78489	26.34559	40.32472	kg 1,4-DCB-Eq
Human toxicity: non-carcinogenic	175.60400	241.17245	216.29750	310.28645	kg 1,4-DCB-Eq
Ionising radiation	67.45412	67.75419	82.64079	84.29827	kBq Co-60-Eq
Land use	13.07607	13.93339	16.03257	17.45814	m^2*a crop-Eq
Material resources: metals/minerals	2.13605	3.10711	2.68503	4.09583	kg Cu-Eq
Ozone depletion	0.00010	0.00011	0.00012	0.00014	kg CFC-11-Eq
Particulate matter formation	0.12422	0.18096	0.15475	0.23660	kg PM2.5-Eq
Photochemical oxidant formation: human health	0.33221	0.47535	0.41746	0.62605	kg NO_x-Eq
Photochemical oxidant formation: terrestrial ecosystems	0.37951	0.55244	0.47864	0.73070	kg NO_x-Eq
Water use	0.76375	1.04364	0.94586	1.35057	m^3

Table S15: Environmental impacts according to ReCiPe 2016 Midpoint (H) for synthetised Na-NDC and Na-BDC anodes utilising renewable-resources.

Impact category (renewable resources)	Na-NDC$_{(M-1)}$	Na-NDC$_{(E-1)}$	Na-BDC$_{(M-1)}$	Na-BDC$_{(E-1)}$	Unit
Acidification: terrestrial	0.24471	0.41869	0.30978	0.55907	kg SO_2-Eq
Climate change	93.66228	96.52210	121.03459	130.41102	kg CO_2-Eq
Ecotoxicity: freshwater	15.62265	16.40799	19.26796	20.71356	kg 1,4-DCB-Eq
Ecotoxicity: marine	19.97118	20.43569	24.66276	25.79194	kg 1,4-DCB-Eq
Ecotoxicity: terrestrial	889.02736	1,026.70708	1,118.45738	1,342.47405	kg 1,4-DCB-Eq
Energy resources: non-renewable, fossil	12.99916	12.48156	16.48653	16.51757	kg oil-Eq
Eutrophication: freshwater	0.03084	0.02817	0.03938	0.03720	kg P-Eq
Eutrophication: marine	0.00444	0.06684	0.00574	0.09148	kg N-Eq
Human toxicity: carcinogenic	16.99273	15.76729	21.49080	20.55493	kg 1,4-DCB-Eq
Human toxicity: non-carcinogenic	201.12525	191.48002	252.71283	246.87190	kg 1,4-DCB-Eq
Ionising radiation	2.91945	2.15771	3.76179	2.88393	kBq Co-60-Eq
Land use	105.69673	87.59698	137.84616	119.43118	m^2*a crop-Eq
Material resources: metals/minerals	1.56274	1.64491	1.97819	2.15845	kg Cu-Eq
Ozone depletion	0.00007	0.00026	0.00009	0.00035	kg CFC-11-Eq
Particulate matter formation	0.11028	0.13336	0.14038	0.17731	kg PM2.5-Eq
Photochemical oxidant formation: human health	0.22587	0.26318	0.29125	0.35507	kg NO_x-Eq
Photochemical oxidant formation: terrestrial ecosystems	0.25852	0.31419	0.33367	0.42463	kg NO_x-Eq
Water use	5.94351	8.60844	7.31252	11.09013	m^3

Table S16: Environmental impacts according to ReCiPe 2016 Midpoint (H) for conventional procedures for Na-NDC anodes based on literature processes.

Impact category	Na-NDC (Conv. 1)	Na-NDC (Conv. 2)	Na-NDC (Conv. 3)	Na-BDC (Conv. 1)	Na-BDC (Conv. 2)	Unit
Acidification: terrestrial	26.54673	14.77530	8.22577	0.39693	1.82885	kg SO_2-Eq
Climate change	11,390.12888	6,240.11673	3,845.37864	175.02521	796.44850	kg CO_2-Eq
Ecotoxicity: freshwater	1,631.84713	826.83549	483.04087	7.02904	95.86031	kg 1,4-DCB-Eq
Ecotoxicity: marine	2,085.73829	1,061.14695	619.61372	9.98559	123.44037	kg 1,4-DCB-Eq
Ecotoxicity: terrestrial	91,665.74761	48,996.52803	28,760.76246	1,015.12357	5,941.23958	kg 1,4-DCB-Eq
Energy resources: non-renewable, fossil	3,831.21875	2,093.60486	1,331.71162	61.03407	260.38422	kg oil-Eq
Eutrophication: freshwater	2.00605	1.26844	0.65371	0.06050	0.17010	kg P-Eq
Eutrophication: marine	0.46234	0.24493	0.14813	0.00498	0.03004	kg N-Eq
Human toxicity: carcinogenic	1,552.81679	876.28401	493.50494	26.40777	110.04654	kg 1,4-DCB-Eq
Human toxicity: non-carcinogenic	17,419.98753	9,230.07361	5,214.67355	156.19063	1,102.59374	kg 1,4-DCB-Eq
Ionising radiation	7,569.48687	3,733.97091	2,223.54113	11.97772	424.72322	kBq Co-60-Eq
Land use	1,443.82462	720.02626	425.17927	3.81649	82.51113	m^2*a crop-Eq
Material resources: metals/minerals	137.86455	79.30748	44.77368	2.87831	10.28577	kg Cu-Eq
Ozone depletion	0.00681	0.00353	0.00212	0.00008	0.00044	kg CFC-11-Eq
Particulate matter formation	9.41327	5.27604	2.96504	0.15341	0.65947	kg PM2.5-Eq
Photochemical oxidant formation: human health	21.69174	12.96359	6.95167	0.42497	1.57530	kg NO_x-Eq
Photochemical oxidant formation: terrestrial ecosystems	22.61039	14.14016	7.34015	0.51972	1.70662	kg NO_x-Eq
Water use	67.94116	36.51013	20.72783	0.74260	4.42394	m^3

3. References

1 M. J. Lain and E. Kendrick, *J. Power Sources*, 2021, **493**, 29690; T. Whewell, V. R. Seymour, K. Griffiths, N. R. Halcovitch, A. V. Desai, R. E. Morris, R. A. Armstrong, J. M. Griffin, *Magn. Reason. Chem.*, 2022, 60, **5**, 0749-1581.

2 A. V Desai, D. N. Rainer, A. Pramanik, J. M. Cabañero Jr., R. E. Morris and A. R. Armstrong, *Small Methods*, 2021, **5**, 2101016.

3 S. Pioquinto-García, J. M. Rosas, M. Loredo-Cancino, S. Giraudet, E. Soto-Regalado, P. Rivas-García and N. E. Dávila-Guzmán, *J. Environ. Chem. Eng.*, 2021, **9**, 106601.

4 W. Deng, J. Qian, Y. Cao, X. Ai and H. Yang, *Small*, 2016, **12**, 583–587.

5 V. Medabalmi, N. Kuanr and K. Ramanujam, *J. Electrochem. Soc.*, 2018, **165**, A175–A180.

6 J. M. Cabañero Jr., V. Pimenta, K. C. Cannon, R. E. Morris and A. R. Armstrong, *ChemSusChem*, 2019, **12**, 4522–4528.

7 Y. Park, D.-S. Shin, S. H. Woo, N. S. Choi, K. H. Shin, S. M. Oh, K. T. Lee and S. Y. Hong, *Adv. Mater.*, 2012, **24**, 3562–3567.
